# Photodynamic priming overcomes platinum resistance from short‐term exposure to select perfluoroalkyl substances in endometrial cancer cell lines

**DOI:** 10.1111/php.14073

**Published:** 2025-03-10

**Authors:** Brittany P. Rickard, Lauren A. Sapienza‐Lundie, Marta Overchuk, Xianming Tan, Victoria L. Bae‐Jump, Melinda S. Yates, Suzanne E. Fenton, Imran Rizvi

**Affiliations:** ^1^ Department of Biological Sciences North Carolina State University Raleigh North Carolina USA; ^2^ Curriculum in Toxicology and Environmental Medicine University of North Carolina School of Medicine, University of North Carolina at Chapel Hill Chapel Hill North Carolina USA; ^3^ Lampe Joint Department of Biomedical Engineering University of North Carolina at Chapel Hill and North Carolina State University Chapel Hill North Carolina USA; ^4^ Department of Biostatistics University of North Carolina School of Public Health Chapel Hill North Carolina USA; ^5^ Lineberger Comprehensive Cancer Center University of North Carolina School of Medicine Chapel Hill North Carolina USA; ^6^ Division of Gynecologic Oncology University of North Carolina at Chapel Hill Chapel Hill North Carolina USA; ^7^ Pathology and Laboratory Medicine University of North Carolina School of Medicine Chapel Hill North Carolina USA; ^8^ Center for Human Health and the Environment North Carolina State University Raleigh North Carolina USA; ^9^ Center for Environmental Health and Susceptibility University of North Carolina at Chapel Hill Chapel Hill North Carolina USA

**Keywords:** endometrial cancer, PDT, PFAS, photodynamic priming, platinum resistance

## Abstract

First‐line treatment for advanced‐stage or recurrent endometrial cancer consists of platinum‐ and taxane‐based chemotherapy, to which many patients will develop resistance. Determining the factors that contribute to platinum resistance and developing alternate treatment options for patients with advanced‐stage gynecologic malignancies is critical to improving survival outcomes. Recently, we published the first study evaluating the contribution of perfluoroalkyl substances (PFAS) exposure to platinum resistance in endometrial cancer cell lines and found that select PFAS induce carboplatin resistance, potentially by dysregulating mitochondrial function. The present study expands upon those findings by examining the efficacy of photodynamic priming (PDP) in combination with carboplatin to overcome PFAS‐induced platinum resistance. Due to the suspected role of mitochondrial dysfunction in platinum resistance, two clinically approved photosensitizers that, in part, localize to mitochondrial membranes or are synthesized in mitochondria were evaluated: benzoporphyrin derivative (BPD) and aminolevulinic acid‐induced protoporphyrin IX (ALA‐PpIX), respectively. Combination of ALA‐PpIX‐mediated PDP + carboplatin resulted in a greater reduction in survival fraction than the same combination with BPD. While PDP with both photosensitizers reduced mitochondrial membrane potential, the reduction was greater with BPD‐PDP than ALA‐PpIX‐PDP. These findings demonstrate that BPD‐PDP and ALA‐PpIX‐PDP in combination with carboplatin can be used to overcome PFAS‐induced platinum resistance in endometrial cancer cells.

## Graphical abstract

PFAS are widespread environmental contaminants that are associated with platinum resistance and mitochondrial membrane hyperpolarization in endometrial cancer cell lines. Photodynamic therapy, using BPD or ALA‐PpIX, in combination with low‐dose carboplatin overcomes PFAS‐induced platinum resistance in endometrial cancer cell lines. A concomitant reduction in mitochondrial membrane potential is also observed.

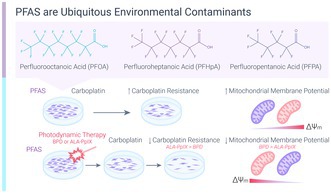

**Abbreviations**
ΔΨm: Mitochondrial membrane potential
ABCG2: ATP‐binding cassette G2
ALA‐PpIX: Aminolevulinic acid‐induced protoporphyrin IX
ATCC: American Type Culture Collection
ATP: Adenosine triphosphate
BPD: Benzoporphyrin derivative
DMSO: Dimethyl sulfoxide
ECACC: European Collection of Authenticated Cell Cultures
EPA: Environmental Protection Agency
FBS: Fetal bovine serum
HEPES: N‐2‐hydroxyethylpiperazine‐N'‐2‐ethanesulfonic acid
JC‐1: 5,5’,6,6’‐tetrachloro‐1,1’3,3’‐tetraethylbenzimidazolocarbo‐cyanine iodide
MEM/EBSS: Minimum Essential Medium with Earle's Balanced Salts
mtDNA: Mitochondrial deoxyribonucleic acid
PDP: Photodynamic priming
PDT: Photodynamic therapy
PFAS: Perfluoroalkyl substances
PFHpA: Perfluoroheptanoic acid
PFOA: Perfluorooctanoic acid
PFPA: Perfluoropentanoic acid

## INTRODUCTION

Endometrial cancer is the most common gynecologic malignancy in developed countries (including the United States) and the 6th most common cancer among women worldwide with over 400,000 new cases diagnosed per year.^1^, ^2^, ^3^ In 2024, an estimated 67,990 women will be diagnosed, and 13,250 will succumb to the disease in the U.S. alone.^4^ Endometrial cancer is among the few cancers for which survival has decreased over the past 40 years, and mortality rates for this disease are increasing more rapidly than any cancer type.^1^, ^5^ While advances in therapeutic regimens have improved survival for some gynecologic malignancies, mortality rates for endometrial cancer have not improved in decades.^6^, ^7^, ^8^ Identifying targetable mechanisms of treatment resistance in endometrial cancer will be critical to reversing these dire trends.

The current standard of care for endometrial cancer is surgery, along with adjuvant radiation therapy in some circumstances.^9^, ^10^ In patients with advanced‐stage disease, which accounts for approximately one‐third of all cases,^11^ or recurrent endometrial cancer, adjuvant platinum‐based chemotherapy, with carboplatin or cisplatin, is often used in combination with taxanes.^10^, ^12^ In some cases, doxorubicin, an anthracycline chemotherapeutic, may be given as either a monotherapy or in combination with a platinum‐based agent.^9^, ^13^ Response rates to these treatments, however, remain poor at approximately 30%.^12^ Targeted therapies, immunotherapy, and combination regimens are additional treatment options for advanced‐stage or recurrent endometrial cancer.^14^, ^15^ It must also be noted that there is a large racial disparity in endometrial cancer outcomes. Black women are more likely to present with advanced‐stage endometrial cancer and higher‐risk histologic subtypes,^16^ contributing to a mortality rate that is double that of Caucasian women.^6^, ^17^

Since advanced‐stage endometrial cancers are increasing in prevalence and the effectiveness of platinum‐based therapies remains limited due to chemoresistance, mechanisms that contribute to platinum resistance must be further explored to elucidate potential therapeutic targets. Increasing evidence shows that mitochondrial dysfunction plays a critical role in both cancer development and chemotherapy resistance.^18^ Our previous studies have demonstrated that environmental exposures, specifically to perfluoroalkyl substances (PFAS), induce platinum resistance in endometrial and ovarian cancer cell lines, potentially by dysregulating mitochondrial function.^9^, ^19^ PFAS are persistent and bioaccumulative chemicals that contaminate drinking water systems throughout the U.S. and worldwide.^20^, ^21^, ^22^ Environmental contamination from PFAS is known to impact the female reproductive system, increasing the risk of endometriosis and causing disruptions in menstrual cyclicity and ovarian hormones, which can ultimately increase cancer risk.^22^, ^23^, ^24^, ^25^ Thus, targeting pathways affected by PFAS in endometrial cancer cells, such as those related to mitochondrial function, could promote cell death through mechanistically distinct pathways from conventional therapies and target chemotherapy failure.

Photodynamic therapy (PDT) is a therapeutic modality that utilizes near‐infrared light and light sensitive molecules, known as photosensitizers, to generate reactive molecular species at the site of activation, including in cancer cells.^26^ Among the preferential sites of localization for benzoporphyrin derivative (BPD), a clinically approved photosensitizer,^27^, ^28^ are mitochondrial membranes, leading to mitochondrial photodamage upon light activation.^27^, ^29^, ^30^, ^31^ Aminolevulinic acid‐induced protoporphyrin IX (ALA‐PpIX, the penultimate product of heme biosynthesis), is also clinically approved and is synthesized, in part, in mitochondria, inducing mitochondrial photodamage following excitation by light.^27^, ^32^ Due to the role of mitochondrial dysfunction in the development of chemoresistance, the use of PDT to sensitize cancer cells to platinum‐based chemotherapy through mitochondrial photodamage warrants exploration in the context of endometrial cancer.^18^, ^33^

PDT is a highly promising treatment modality for gynecologic malignancies. Studies have shown that PDT synergizes with platinum‐based chemotherapy in 3D and in vivo models of ovarian cancer.^33^, ^34^, ^35^ More recent studies have also demonstrated the efficacy of photodynamic priming (PDP), a sub‐tumoricidal variant of PDT, in overcoming platinum resistance arising from PFAS exposure in ovarian cancer cells, potentially by diminishing mitochondrial membrane potential (ΔΨ_m_).^27^ Limited clinical studies have also demonstrated promising PDT efficacy as a fertility‐sparing treatment in women with low‐grade and early‐stage endometrial cancer who wish to avoid hysterectomy.^36^, ^37^

The current study describes, for the first time, the use of PDP to overcome PFAS‐induced platinum resistance in endometrial cancer cells. PDP efficacy using two clinically relevant photosensitizers, BPD and ALA‐PpIX, was evaluated in HEC‐1B and Ishikawa cell lines. First, the impact of exposure to PFAS and PFAS mixtures on PDT efficacy was evaluated. In both cell lines, only minor impacts were noted following PFAS exposure, indicating minimal interference of PFAS with PDT response. Next, combinations using either BPD‐PDP or ALA‐PpIX‐PDP and carboplatin were evaluated as a means of overcoming platinum resistance in endometrial cancer cells. Both BPD and ALA‐PpIX enhanced response to chemotherapy, particularly among the lower carboplatin doses tested. Finally, the effects of combination therapy on ΔΨ_m_ were evaluated. Combination therapy using BPD‐PDP + carboplatin reduced ΔΨ_m_ in PFAS‐exposed endometrial cancer cells to a greater extent than ALA‐PpIX‐mediated PDP.

## METHODS

### Cell culture

As described previously,^9^ human endometrial adenocarcinoma HEC‐1B and Ishikawa cell lines were obtained from the American Type Culture Collection (ATCC) and the European Collection of Authenticated Cell Cultures (ECACC), respectively. Culture medium for HEC‐1B cells was McCoy's 5A medium (ATCC, Manassas, VA, USA) supplemented with 10% fetal bovine serum (FBS, Cytiva HyClone™, Marlborough, MA, USA), 2 mM l‐glutamine (Corning, Corning, NY, USA), and 1% antibiotic‐antimycotic solution (Corning). Culture medium for Ishikawa cells was minimum essential medium with Earle's balanced salt solution (MEM/EBSS, Cytiva HyClone™) supplemented with 5% FBS, 1% nonessential amino acids (Gibco, Billings, MT, USA), 2 mM l‐glutamine, 100 U/mL penicillin, and 100 mg/mL streptomycin (Sigma‐Aldrich, St. Louis, MO, USA). Cells were grown at 37°C in a humidified incubator with 5% CO_2_ and routinely tested for mycoplasma contamination using the MycoAlert™ PLUS Kit (Lonza Bioscience, Basel, Switzerland, Catalog #LT07‐710). Cells were grown through passage 30, when a new batch of frozen cells was thawed and grown. The HEC‐1B cell line was authenticated by the Virology Core at the University of North Carolina at Chapel Hill using Ion Torrent Precision ID GlobalFiler™ Next Generation Sequencing Short Team Repeat Panel (Applied Biosystems, Waltham, MA, USA). The Ishikawa cell line was authenticated by ECACC before arrival.

### PFAS stock preparation

PFAS stock solutions were prepared as described previously.^9^, ^19^, ^27^ PFOA (CAS#335‐67‐1) was purchased from Synquest Laboratories (Alachua, FL, USA, Catalog #2121‐3‐18, 98% purity), PFHpA (CAS#375‐85‐9) was purchased from Sigma‐Aldrich (Catalog #342041‐5G, 97% purity), and PFPA was purchased from TCI America (Portland, OR, USA, Catalog #N06055G, 98% purity). Using 1.0 N potassium hydroxide in methanol, 10 mM stocks of each chemical were prepared (Lab Chem Inc., Zelienople, PA, USA, Catalog #LC195402; referred to as “vehicle”). PFAS stock solutions were stored at −20°C. *N*‐2‐hydroxyethylpiperazine‐*N*′‐2‐ethanesulfonic acid (HEPES) was added to cell culture medium to limit drastic pH changes from either the addition of acidic PFAS or alkaline potassium hydroxide in methanol (vehicle).

### Photosensitizer stock preparation

Stock solutions of BPD and ALA were prepared as described in previous publications.^27^ A stock solution of 165.75 mM ALA hydrochloride (Sigma‐Aldrich, Catalog #A7793‐500MG) was prepared by dissolving 500 mg of ALA in 10 mL unbuffered, cell culture grade water. This stock was directly added to culture medium to prepare dosing concentrations of ALA (wrapped in foil to protect from light). Stock solutions of ~450 μM BPD (Sigma‐Aldrich, Catalog #SML0534) were prepared by dissolving 5.39 mg BPD in 25 mL dimethyl sulfoxide. BPD stock solutions were then aliquoted (200 μL aliquots) and stored at −20°C until use. BPD concentrations in each aliquot were confirmed using either the QuickDrop (Molecular Devices, San Jose, CA, USA) or the Cary 60 UV–vis Spectrophotometer (Agilent Technologies, Santa Clara, CA, USA) prior to each use.

### Measurement of baseline PDT efficacy

HEC‐1B and Ishikawa cells were seeded at densities of 2500 cells/well in white‐walled, clear‐bottom 96‐well plates (at least one technical replicate per condition, at least three biological replicates). Seeding densities were based on previous studies showing the optimal seeding density for a 6‐day experiment was 2500 cells/well based on the linear dynamic range of the CellTiter Glo Luminescent Cell Viability Assay (Promega Corp., Madison, WI, USA).^9^ To determine the efficacy of BPD and ALA‐PpIX for PDT, cells were seeded into plates and allowed to grow for 24 h prior to receiving a medium change. Experimental timelines were consistent with PFAS exposure experiments described in the next subsection. Medium was removed after 48 h and cells were exposed to 0.25 μM BPD or 1 mM ALA‐containing medium for 90 min or 4 h, respectively.^27^ Concentrations of photosensitizers were selected based on previous studies.^27^, ^38^, ^39^ Photosensitizer‐containing medium was removed and replaced with fresh culture medium. Plates were then exposed to light (BPD: 690 nm or ALA‐PpIX: 630 nm) using an LED‐based irradiation platform (LEDBox, BioLambda, Sao Paulo, Brazil): BPD‐PDT in HEC‐1B cells: 0.03–1 J/cm^2^ at 20.12 mW/cm^2^ and Ishikawa cells: 0.01–0.33 J/cm^2^ at 6.71 mW/cm^2^ (Figure S1); ALA‐PpIX‐PDT in HEC‐1B and Ishikawa cells: 0.1–5 J/cm^2^ at 46.3 mW/cm^2^. Irradiation times for each photosensitizer were set by a controller connected to the LED platform (BlackBox Smart, BioLambda) and verified independently to deliver appropriate energy densities. After PDT, plates were returned to the incubator for an additional 48 h prior to evaluating survival fraction using the CellTiter Glo assay. After acclimating each plate to room temperature for 30 min, 50 μL medium was removed from each well and 50 μL CellTiter Glo reagent was added. Plates were orbitally shaken for 2 min in the SpectraMax iD3 plate reader (Molecular Devices, San Jose, CA, USA) or the CLARIOstar Plus (BMG Labtech, Cary, NC, USA) then allowed to equilibrate at room temperature for 10 min. After the signal was allowed to stabilize, luminescence of each plate was read using the plate reader.

### Measurement of PDT efficacy in PFAS‐exposed cells

HEC‐1B and Ishikawa cells were seeded at 2500 cells/well and allowed to grow in complete medium for 24 h prior to being exposed to PFAS (two technical replicates per condition, at least three biological replicates). Dosing solutions of PFOA, PFHpA, PFPA, or mixtures of these chemicals were made in serum‐free medium and 2X serum‐containing medium for each cell line. Following previously published dosing schemes,^9^, ^19^, ^27^ cells were either exposed to 0.5 or 2 μM PFOA, PFHpA, or PFPA in 50 μL serum‐free medium for 1 h prior to incubation with 50 μL of the same chemical or mixture in 2X serum‐containing medium (final well volume = 100 μL). Cells remained in the resulting medium for an additional 47 h (total exposure time = 48 h). The same dosing scheme as above was used for PFAS mixtures. HEC‐1B and Ishikawa cells exposed to PFAS mixtures were exposed to combinations of PFOA + PFHpA, PFOA + PFPA, PFHpA + PFPA, or PFOA + PFHpA + PFPA. The resulting concentration of mixtures of two chemicals was 2 μM (1 + 1 μM of each) or 2.25 μM (0.75 + 0.75 + 0.75 μM) for the mixture of three chemicals. For all experiments described, vehicle exposure groups received 1% potassium hydroxide in methanol following the above dosing scheme. Following 48‐h exposures to vehicle, PFAS, or PFAS mixtures, medium was then removed from each well and replaced with 100 μL complete medium containing BPD or ALA. BPD was dosed at a concentration of 0.25 μM and allowed to incubate with cells for 90 min, while ALA was dosed at 1 mM and incubated with cells for 4 h. After each photosensitizer's respective incubation period, photosensitizer‐containing medium was removed from each well and replaced with 50 μL complete medium. BPD‐exposed cells were irradiated as described above at light doses ranging from 0.03 to 1 J/cm^2^ (HEC‐1B) or 0.01 to 0.33 J/cm^2^ (Ishikawa). For ALA‐exposed cells, both cell lines were irradiated as described above at light doses ranging from 0.1 to 5 J/cm^2^. Following irradiation, 50 μL of fresh medium was added to each well and plates were returned to the incubator. After 48 h, survival fraction was measured using the CellTiter Glo assay as described above. For all photosensitizer incubation steps, plates were covered in foil during transport and dosed in dark environments to protect photosensitive cells from supplementary light.

### Evaluation of combination treatment efficacy (PDP + carboplatin)

HEC‐1B and Ishikawa cells were seeded and exposed to PFAS following the timeline and method in the previous subsection (two technical replicates per condition, at least three biological replicates). After 48 h of PFAS exposure, 0.25 μM BPD or 1 mM ALA was added to appropriate wells in complete medium for 90 min or 4 h, respectively. After the respective incubation period, photosensitizer‐containing medium was removed from plates, and fresh culture medium was added prior to irradiation. Since a goal of PDP is to minimize toxicity, only light doses resulting in minimal to no significant decreases in survival fraction were selected for each photosensitizer and cell line. BPD‐exposed HEC‐1B cells were irradiated using the 690 nm LED box at an irradiance of 20.12 mW/cm^2^ and light dose of 0.03 J/cm^2^ (~1 s). Due to their increased sensitivity to BPD‐PDT, BPD‐exposed Ishikawa cells were irradiated using the 690 nm LED box at an irradiance of 6.71 mW/cm^2^ and light dose of 0.01 J/cm^2^ (~1 s). ALA‐exposed HEC‐1B and Ishikawa cells were irradiated using the 630 nm LED box at an irradiance of 46.3 mW/cm^2^ and light dose of 0.1 J/cm^2^ (~3 s). After irradiation, cells were treated with chemotherapy, in this case carboplatin, for a total of 48 h. For HEC‐1B cells, an optimal range of carboplatin doses was determined to be 50–400 μM while Ishikawa cells tolerated 100–800 μM.^9^ Stocks were prepared by dissolving 1.8563 mg carboplatin in 1 mL cell culture medium to achieve a 5 mM stock concentration. Solutions ranging from 100 to 400 μM (HEC‐1B) or 200 to 800 μM (Ishikawa) were then prepared for dosing. Concentration ranges were doubled since 50 μL complete medium was added to plates prior to irradiating, and 50 μL carboplatin‐containing medium was added to each well (e.g., 50 μL complete medium + 50 μL 800 μM carboplatin = 400 μM carboplatin). After 48 h of carboplatin treatment, survival fraction was evaluated using the CellTiter Glo assay as described above.

### Measurement of ΔΨ_m_ after exposure to PFAS, treatment with PDP, and/or treatment with combination therapy

Cells were seeded at a density of 20,000 cells/well in 96‐well, black‐walled, clear‐bottom plates and allowed to grow for 24 h (two technical replicates per condition, at least three biological replicates). This seeding density was based on previous studies, where 40,000 cells/well was used for ovarian cancer cells that were typically seeded at 5000 cells/well.^9^, ^19^, ^27^ After 24 h, medium was removed from plates and HEC‐1B and Ishikawa cells were simultaneously exposed to PFAS and photosensitizers in serum‐free medium. Serum‐free medium was used to ensure adequate cell uptake of both PFAS and BPD or ALA. Since PFAS and photosensitizers were being exposed concurrently, PFAS exposure concentrations were increased to 1 or 4 μM, BPD concentrations were increased to 0.5 μM, and ALA concentrations were increased to 2 mM (e.g., 4 μM PFAS in 50 μL medium + 0.5 μM BPD in 50 μL medium = 2 μM PFAS + 0. 25 μM BPD in a total of 100 μL medium). After either 90 min (BPD) or 4 h (ALA) of simultaneous PFAS and photosensitizer exposure, medium was removed, and complete medium was added to each well. Plates were then irradiated following the PDP dosing schemes in the above section. After irradiation, 10 μg/mL 5,5′,6,6′‐tetrachloro‐1,1′3,3′‐tetraethylbenzimidazolocarbo‐cyanine iodide (JC‐1) dye (Invitrogen, Waltham, MA, USA) was added to each well for 15 min. This was performed after irradiation to prevent dye photobleaching. After 15 min, JC‐1‐containing medium was removed from wells and cells were washed with PBS prior to treatment with carboplatin. Carboplatin was dosed at 50–200 μM for HEC‐1B cells and 100–400 μM for Ishikawa cells in complete culture medium for 1 h. Although concentrations of 400 and 800 μM were maximum doses for HEC‐1B and Ishikawa cells, respectively, a goal of combination therapy is to use lower doses of carboplatin. After 1 h of chemotherapy treatment, the JC‐1 red:green aggregate ratio was evaluated using a fluorescence protocol on the SpectraMax iD3 plate reader (green aggregate – excitation: 488 nm, emission: 529 nm; red aggregate – excitation: 488 nm, emission: 590 nm).

### Statistical analysis

To examine the effects of PFAS concentration, PDT light dose, photosensitizer, and carboplatin concentration on survival fraction and ΔΨ_m_, unpaired *t*‐tests and two‐way ANOVA were employed as appropriate. All tests are two‐sided at alpha level 0.05 unless otherwise specified. All analyses were performed in Prism 10.0 software (GraphPad, San Diego, CA, USA) or R Statistical Software (v4.1.1; R Core Team 2021).^40^

## RESULTS

### PFAS exposure alters PDT efficacy at select light doses in HEC‐1B and Ishikawa cells

The goal of using PDP in the present study was to overcome platinum resistance resulting from PFAS exposure in endometrial cancer cells with lower doses of carboplatin to enhance efficacy while mitigating toxicity; however, we first needed to identify light doses compatible with PDP in PFAS‐exposed cells. Our previous studies have demonstrated that no absorbance is observed by PFAS at the wavelengths of light used for BPD‐PDT and ALA‐PpIX‐PDT.^27^ Thus, the impact of PFAS exposure on PDT response in endometrial cancer cells was evaluated. For BPD‐PDT, energy densities between 0.03 and 1.0 J/cm^2^ at an irradiance of 20.12 mW/cm^2^ were evaluated in HEC‐1B cells (Figure S2A). Using the same dose parameters in Ishikawa cells resulted in substantial reductions in survival fraction at the lowest energy densities and near complete killing by 0.1 J/cm^2^ (irradiation time: 5 s; Figure S1). Based on this dose–response relationship, the irradiance was reduced to 6.71 mW/cm^2^ for PDT in Ishikawa cells in order to evaluate response to a range of lower energy densities: 0.01‐0.33 J/cm^2^ (Figure S2B). For ALA‐PpIX PDT, energy densities between 0.1 and 5.0 J/cm^2^ at an irradiance of 46.3 mW/cm^2^ were evaluated in both HEC‐1B and Ishikawa cells (Figure S2C,D). To understand the impact of PFAS exposure on response to either BPD‐PDT or ALA‐PpIX‐PDT, HEC‐1B and Ishikawa cells were exposed to 0.5 or 2 μM PFOA and PFHpA prior to PDT.

#### BPD‐PDT

**FIGURE 1:**
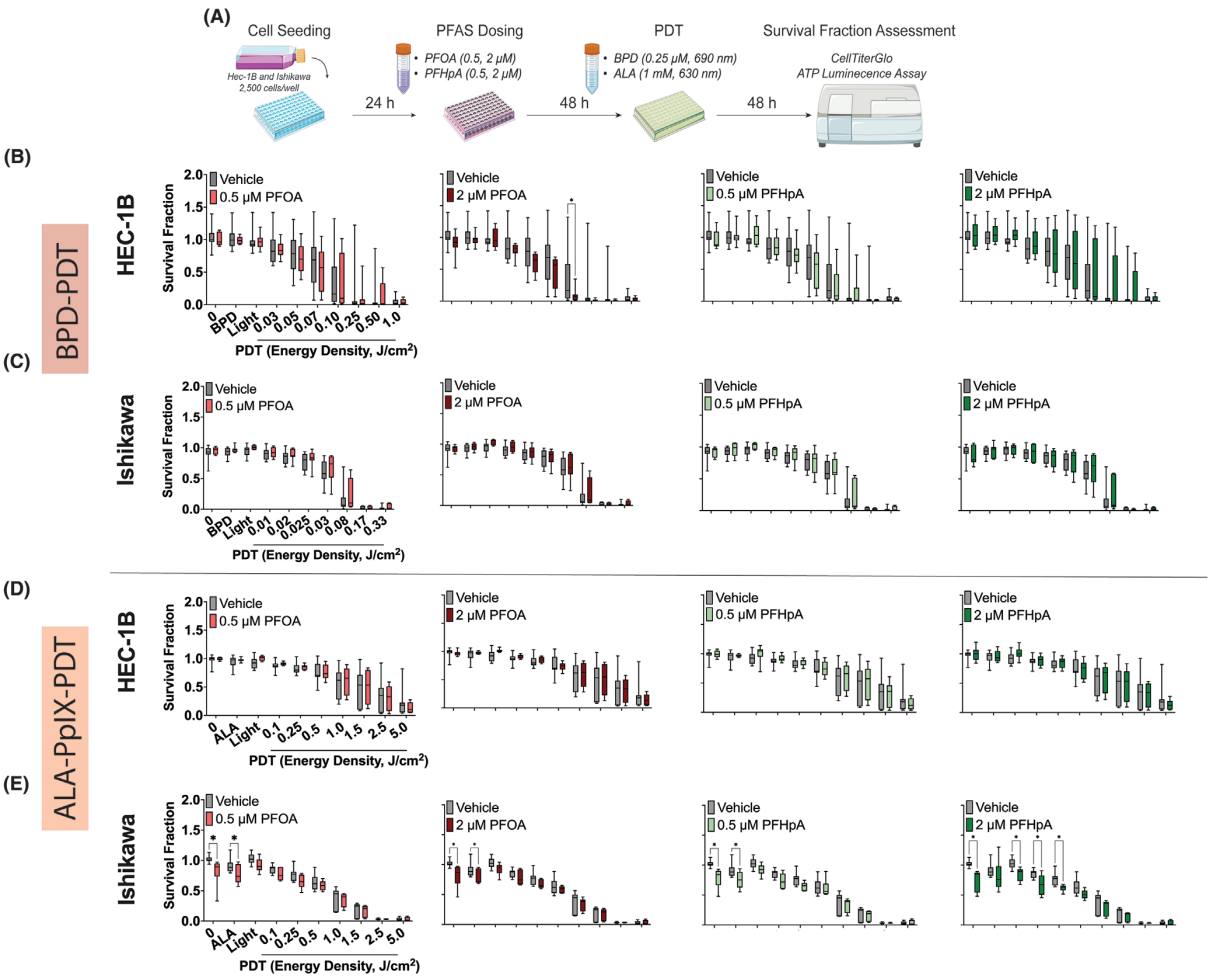
Efficacy of BPD‐PDT (B,C) and ALA‐PpIX‐PDT (D,E) in HEC‐1B and Ishikawa cells exposed to individual PFAS. (A) Timeline of experiments. Dose responses of BPD‐PDT in (B) HEC‐1B cells exposed to PFOA and PFHpA or (C) Ishikawa cells exposed to PFOA and PFHpA. Dose responses of ALA‐PpIX‐PDT in (D) HEC‐1B cells exposed to PFOA and PFHpA or (E) Ishikawa cells exposed to PFOA and PFHpA. Data shown are normalized to their own no treatment control and are from *n* = at least three independent experiments with at least one technical replicate each. Significant differences between PFAS‐exposed groups and vehicle‐exposed groups are denoted by * (*p* < 0.05) and were determined using a two‐way ANOVA with Dunnett's test for multiple comparisons. Contains Servier Medical Art stock images provided by Servier, licensed under a Creative Commons Attribution 3.0 unported license.

In Figure 1, BPD‐PDT dose–response curves are shown for HEC‐1B and Ishikawa cells following PFOA or PFHpA exposure. Survival fraction in the PFAS exposed groups is compared to the vehicle control, which was 1% potassium hydroxide in methanol in complete culture medium. In HEC‐1B cells, no significant differences in BPD‐PDT dose response were observed in vehicle‐exposed cells compared to untreated controls (Figure S3A). For most PFAS exposure groups in HEC‐1B cells, no significant difference was observed compared to the vehicle only group, indicating that short‐term exposure to PFAS does not affect PDT efficacy. A significant reduction in survival fraction was only observed in HEC‐1B cells exposed to 2 μM PFOA then treated with a light dose of 0.1 J/cm^2^ (0.09 ± 0.11, *p* = 0.014, Figure 1B) compared to the vehicle‐only group for the same light dose (0.38 ± 0.45). Interestingly, a significant decrease in survival fraction was also observed at this light dose in 2 μM PFPA‐exposed cells (Figure S4A).

Ishikawa cells responded similarly when exposed to PFOA and PFHpA followed by treatment with BPD‐PDT. No significant differences in survival were observed in any exposure group compared to controls, indicating that short‐term PFAS exposure does not significantly affect BPD‐PDT efficacy (Figure 1C). This also held true in Ishikawa cells that were exposed to PFPA (Figure S4C). Although no instances of altered survival fraction were observed in PFAS exposure groups compared to the vehicle control, significant decreases in survival fraction post‐BPD‐PDT were observed in vehicle‐exposed cells compared to unexposed controls (Figure S3C). Interestingly, this effect was only observed in Ishikawa cells treated with BPD‐PDT. No significant vehicle‐induced effects of survival fraction were observed in HEC‐1 cells or in Ishikawa cells treated with ALA‐PpIX‐PDT. This may indicate interference of BPD‐PDT by methanol toxicity in Ishikawa cells and warrants further exploration, especially considering the heightened sensitivity of Ishikawa cells to BPD‐PDT (Figure S1). Additionally, it is important to note that in some cases, survival fraction significantly decreased in Ishikawa cells receiving photosensitizer only (no light). This could be due to a dark effect of the photosensitizer (i.e., toxicity in the absence of light), absorption of blue light present in ambient room lighting (although standard precautions were taken to control for this), or residual light carryover from adjacent wells receiving irradiation (Figures 1, S1, and S3).

#### ALA‐PpIX‐PDT

Similar to the results observed with BPD‐PDT, response to ALA‐PpIX‐PDT was not significantly modified by short‐term exposure to either PFOA, PFHpA, or PFPA in HEC‐1B cells (Figures 1D and S4B). While no significant changes in survival fraction were observed in HEC‐1B cells, a significant reduction in survival fraction was observed in Ishikawa cells exposed to 2 μM PFHpA, but not PFOA, then treated with light doses of 0.1 J/cm^2^ (0.67 ± 0.17, *p* = 0.003) and 0.25 J/cm^2^ (0.62 ± 0.06, *p* = 0.03) compared to corresponding vehicle only controls (0.1 J/cm^2^: 0.85 ± 0.07, 0.25 J/cm^2^: 0.76 ± 0.10; Figure 1E). A similar decrease in survival fraction was also observed in Ishikawa cells exposed to 2 μM PFPA then treated with a light dose of 0.1 J/cm^2^ (0.70 ± 0.07, *p* = 0.006, Figure S4D). These results suggest that short‐term PFAS exposure may increase the sensitivity of Ishikawa, but not HEC‐1B, cells to ALA‐PpIX‐PDT at specific light doses.

### BPD‐PDT and ALA‐PpIX‐PDT dose responses are not altered significantly by exposure to PFAS mixtures in HEC‐1 or Ishikawa cells

Compared to individual PFAS chemicals, PFAS mixtures are more human‐relevant since complex mixtures of tens or hundreds of these chemicals may be present in drinking water supplies. Thus, the effects of mixtures of PFOA + PFHpA, PFOA + PFPA, PFHpA + PFPA, and PFOA + PFHpA + PFPA on PDT efficacy were explored in both endometrial cancer cell lines. Although chemotherapy resistance was not observed in either cell line following short‐term exposure to PFAS mixtures,^9^ results from cells exposed to PFHpA + PFPA and PFOA + PFHpA + PFPA are presented due to our previous study demonstrating that these mixtures induce chemoresistance in ovarian cancer cells.^19^

#### BPD‐PDT

**FIGURE 2:**
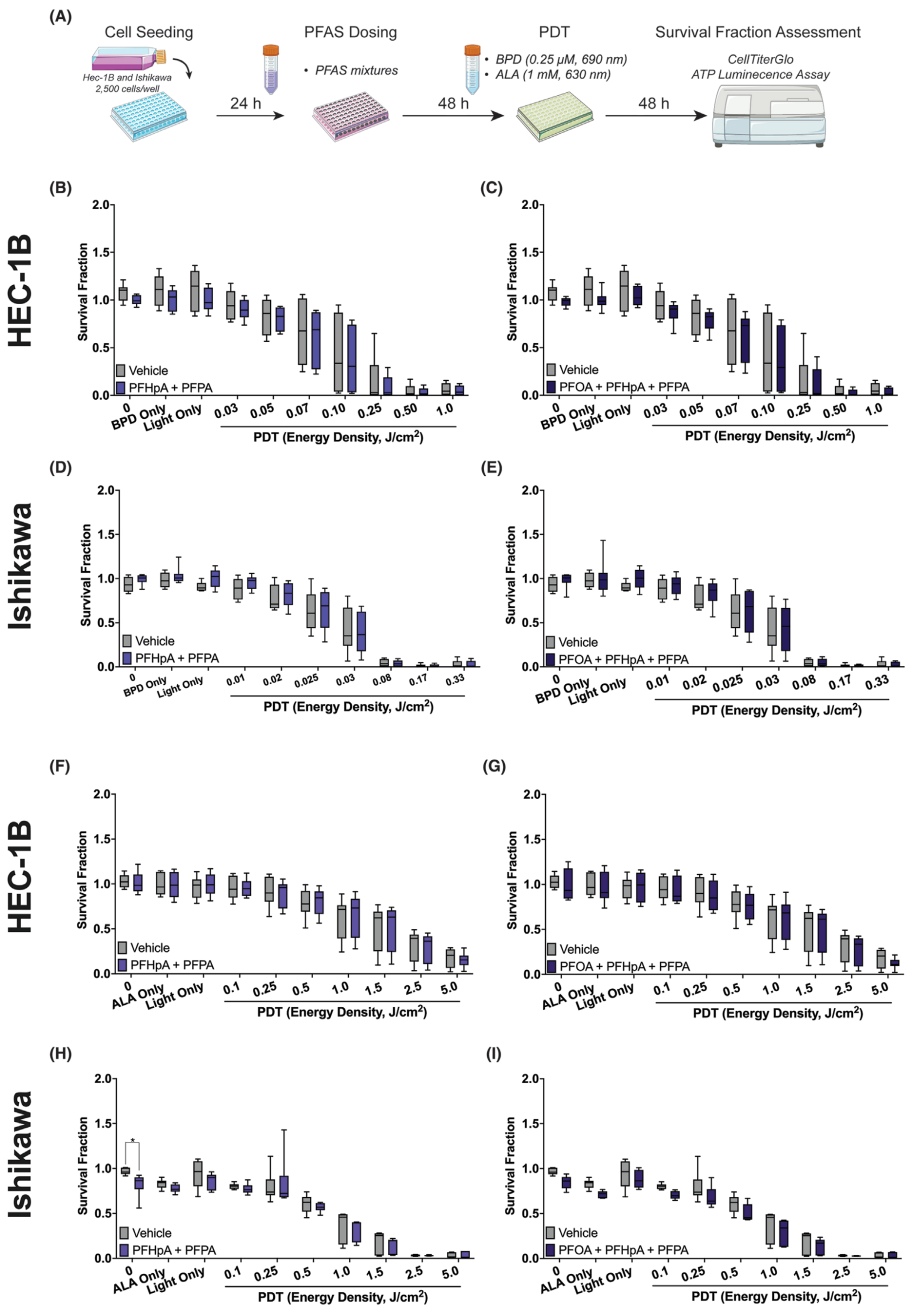
Efficacy of BPD‐PDT (B–E) and ALA‐PpIX‐PDT (F–I) in HEC‐1B and Ishikawa cells exposed to PFAS mixtures. (A) Timeline of experiments. Dose responses of BPD‐PDT in HEC‐1B cells exposed to (B) PFHpA + PFPA and (C) PFOA + PFHpA + PFPA or Ishikawa cells exposed to (D) PFHpA + PFPA and (E) PFOA + PFHpA + PFPA. Dose responses of ALA‐PpIX‐PDT in HEC‐1B cells exposed to (F) PFHpA + PFPA and (G) PFOA + PFHpA + PFPA or Ishikawa cells exposed to (H) PFHpA + PFPA and (I) PFOA + PFHpA + PFPA. Data shown are normalized to their own no treatment control and are from *n* = at least three independent experiments with two technical replicates each. Contains Servier Medical Art stock images provided by Servier, licensed under a Creative Commons Attribution 3.0 unported license.

Compared to individual PFAS chemicals, for which instances of significant reductions in survival fraction were observed following BPD‐PDT in HEC‐1B and/or Ishikawa cells, no significant effects of PFAS mixtures were observed in either HEC‐1B or Ishikawa cells after exposure to PFHpA + PFPA or PFOA + PFHpA + PFPA (Figure 2B–E). Importantly, no significant changes in survival fraction were observed in HEC‐1B or Ishikawa cells exposed to PFOA + PFHpA or PFOA + PFPA either (Figures S5A–D), suggesting that PFAS mixtures do not interfere with BPD‐PDT efficacy.

While no significant changes in survival fraction were observed in Ishikawa cells exposed to PFAS and treated with BPD‐PDT, it is notable that unexposed Ishikawa cells were more sensitive to BPD‐PDT than HEC‐1B cells (Figure S1). When Ishikawa cells were irradiated with 20.12 mW/cm^2^ light doses ranging from 0.03 to 1 J/cm^2^ (the range of light doses used for HEC‐1B cells in the present study and ovarian cancer cells in previous studies^27^), survival fraction decreased by ~70% at the lowest light dose (~1 s, Figure S1A). This indicates that, compared to HEC‐1B cells, BPD‐PDT is much more potent at baseline in Ishikawa cells. Interestingly, when Ishikawa cells were exposed to PFAS or PFAS mixtures prior to irradiation at these cytotoxic light doses, survival fraction decreased further across various exposure groups (Figure S1B–E), indicating that PFAS exposure may enhance BPD‐PDT potency in Ishikawa cells at the higher energy densities evaluated in the present study. Thus, to achieve feasible energy densities for BPD‐PDP, the irradiance was adjusted in Ishikawa cells (6.71 mW/cm^2^).

#### ALA‐PpIX‐PDT

No significant changes in survival fraction were observed in either cell line after exposure to PFAS mixtures followed by treatment with ALA‐PpIX‐PDT (Figure 2F–I). This was also true in cells exposed to PFOA + PFHpA or PFOA + PFPA (Figure S5E–H), further suggesting that exposure to PFAS mixtures does not impact PDT efficacy in HEC‐1B or Ishikawa cells.

### BPD‐PDP and ALA‐PpIX‐PDP overcome PFAS‐induced carboplatin resistance in endometrial cancer cell lines

**FIGURE 3:**
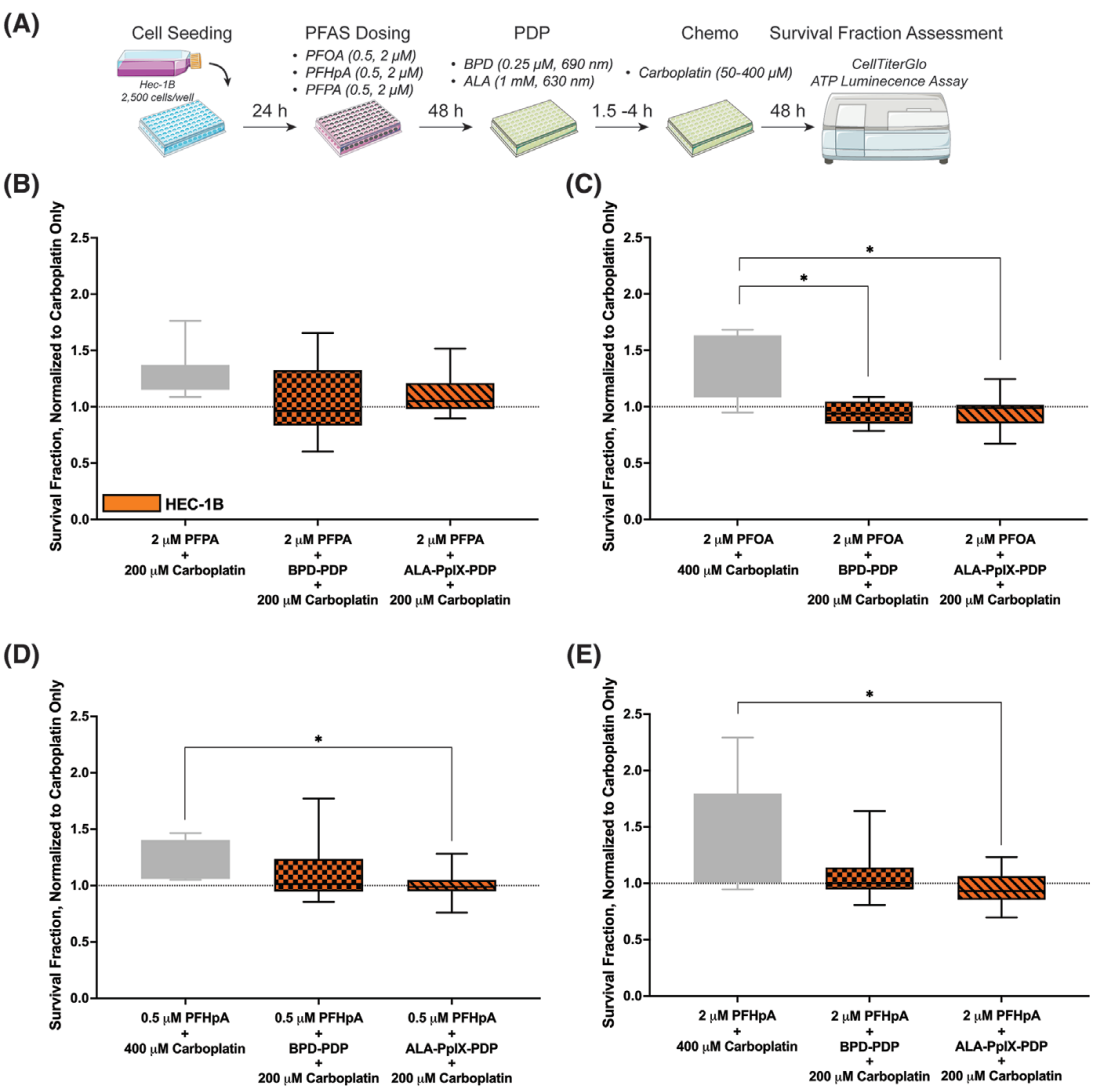
Evaluation of combination therapy using either BPD‐PDP or ALA‐PpIX‐PDP and carboplatin to overcome platinum resistance in HEC‐1B cells exposed to PFAS. (A) Timeline of experiments. Effects of combination therapy using BPD‐PDP (*hν*: 0.03 J/cm^2^) or ALA‐PpIX‐PDP (*hν*: 0.1 J/cm^2^) with carboplatin on survival fraction of HEC‐1B cells in PFAS exposure groups where platinum resistance was observed. These included (B) 2 μM PFPA + 200 μM carboplatin, (C) 2 μM PFOA + 400 μM carboplatin, (D) 0.5 μM PFHpA + 400 μM carboplatin, and (E) 2 μM PFHpA + 400 μM carboplatin. Gray bars shown represent previously published data^9^; remaining data shown are normalized to the vehicle control for each respective chemotherapy concentration and are from *n* = at least 3 independent experiments with two technical replicates each. Significant differences between combination therapy‐treated exposure groups and exposure groups treated only with carboplatin are denoted by * (*p* < 0.05) and were determined using unpaired *t*‐tests. Contains Servier Medical Art stock images provided by Servier, licensed under a Creative Commons Attribution 3.0 unported license.

Based on BPD‐PDT and ALA‐PpIX‐PDT dose responses shown in Figures 1 and 2, BPD‐PDP and ALA‐PpIX‐PDP were performed using light doses below the threshold of toxicity. For BPD‐PDP, 0.03 and 0.01 J/cm^2^ were used for HEC‐1B cells (0.99 ± 0.08) and Ishikawa cells (1.04 ± 0.06), respectively. For ALA‐PpIX‐PDP, 0.1 J/cm^2^ was used for both cell lines (HEC‐1B: 0.95 ± 0.06; Ishikawa: 0.78 ± 0.09). For this study, PDP is defined as PDT at a dose that exerts less than 25% cell killing. Since one of the goals of incorporating PDP in our studies was to use lower carboplatin doses, only 50–200 or 100–400 μM were used for HEC‐1B and Ishikawa cells, respectively. These chemotherapy ranges do not include the highest concentration used for dose–response experiments (HEC‐1B: 400 μM, Ishikawa: 800 μM).^9^ The effects of PDP in combination with carboplatin were compared to data from our previously published study,^9^ illustrating survival fraction in HEC‐1B cells exposed to PFAS followed by treatment with carboplatin (without PDP). These data are represented with gray bars in Figure 3. Carboplatin resistance was observed in the following groups in HEC‐1B cells: 2 μM PFPA + 200 μM carboplatin, 0.5 or 2 μM PFHpA +400 μM carboplatin, and 2 μM PFOA + 400 μM carboplatin.^9^

#### BPD‐PDP

In each group where platinum resistance was previously reported for HEC‐1B cells,^9^ survival fraction was reduced by BPD‐PDP in combination with carboplatin, albeit not significantly for every group. A significant decrease in survival fraction was observed in the group exposed to 2 μM PFOA followed by treatment with combination BPD‐PDP + 200 μM carboplatin (0.95 ± 0.11), compared to the same group without PDP (1.36 ± 0.28, *p* < 0.001; Figure 3C). Overall numerical trends toward a decrease in survival fraction were observed in other groups: (i) 2 μM PFPA followed by BPD‐PDP + 200 μM carboplatin (1.05 ± 0.34) relative to the same group without PDP (1.28 ± 0.24, Figure 3B), (ii) 0.5 μM PFHpA then BPD‐PDP + 200 μM carboplatin (1.14 ± 0.33) versus without PDP (1.26 ± 0.17, Figure 3D), and (iii) 2 μM PFHpA then BPD‐PDP + 200 μM carboplatin (1.07 ± 0.24, Figure 3E) compared to 1.42 ± 0.50 without PDP. These results are noteworthy given that most instances of chemoresistance in HEC‐1B cells were observed at 400 μM carboplatin, whereas BPD‐PDP + 200 μM carboplatin reduced survival fraction to a comparable extent or even greater.

#### ALA‐PpIX‐PDP

Compared to BPD‐PDP, combination ALA‐PpIX‐PDP + carboplatin resulted in more instances of significant reduction in survival fraction in HEC‐1B cells, relative to corresponding platinum resistant groups without PDP. Significant decreases in survival fraction were observed in most groups: 2 μM PFOA + 200 μM carboplatin (chemo only: 1.36 ± 0.28, combination: 0.96 ± 0.15, *p* = 0.002), 0.5 μM PFHpA + 200 μM carboplatin (chemo only: 1.26 ± 0.17, combination: 0.99 ± 0.13, *p* = 0.003), and 2 μM PFHpA + 200 μM carboplatin (chemo only: 1.42 ± 0.50, combination: 0.95 ± 0.16, *p* = 0.02; Figure 3). A decrease, albeit not significant, in survival fraction was also observed in the 2 μM PFPA + 200 μM carboplatin group (*p* = 0.06). These data highlight the value of PDP, particularly ALA‐PpIX‐PDP, to overcome PFAS‐induced chemoresistance and to enable the use of lower doses of carboplatin in the treatment of endometrial cancer cells.

### Low‐dose carboplatin in combination with BPD‐PDP or ALA‐PpIX‐PDP is most effective at overcoming PFAS‐induced platinum resistance in endometrial cancer cell lines

**FIGURE 4:**
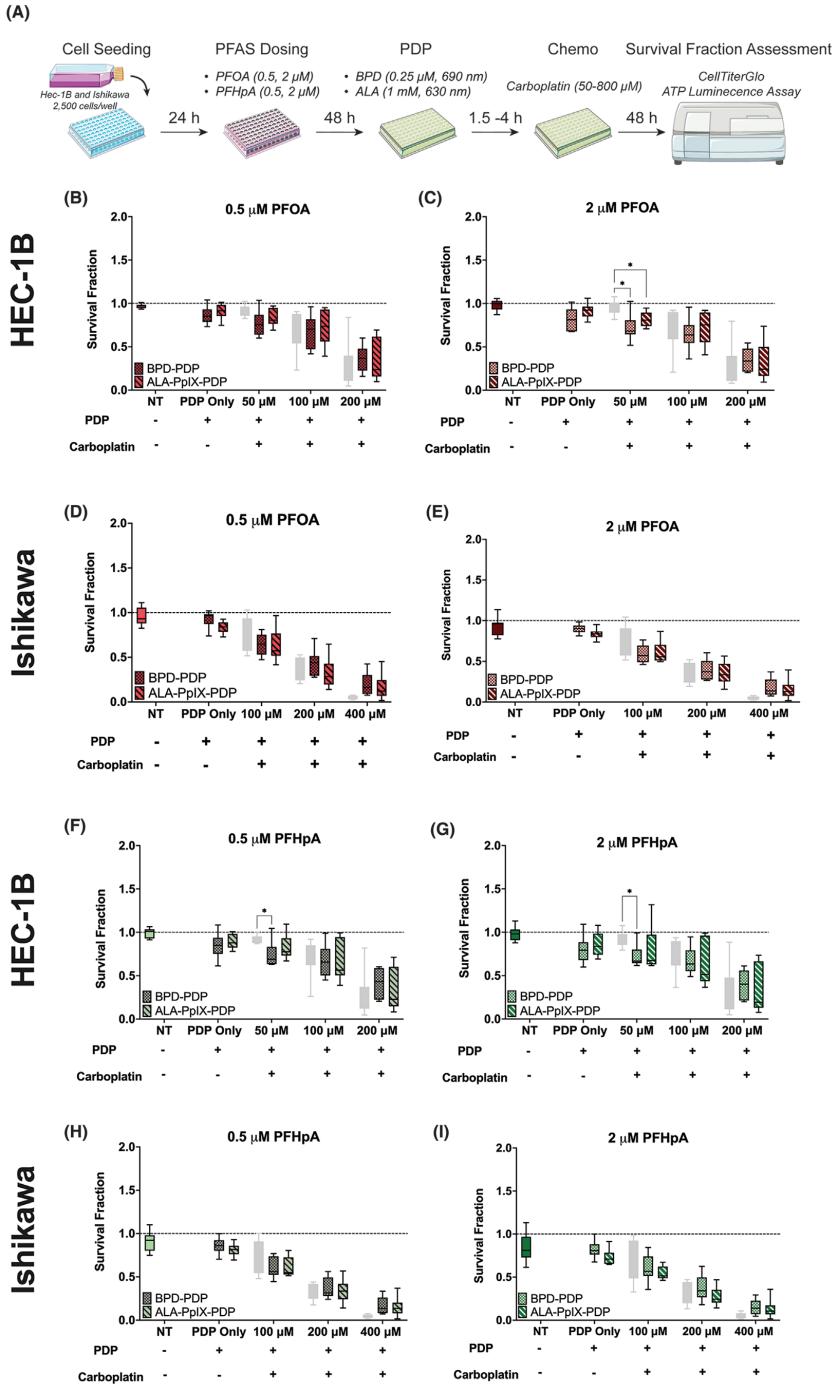
Comparison of survival fraction in HEC‐1B and Ishikawa cells exposed to PFAS then treated with carboplatin only versus combination therapy. (A) Timeline of experiments. Dose responses of HEC‐1B cells exposed to (B, C) PFOA or (F, G) PFHpA then treated with carboplatin only (gray bars), BPD‐PDP (*hν*: 0.03 J/cm^2^) + carboplatin (checkered bars) or ALA‐PpIX‐PDP (*hν*: 0.1 J/cm^2^) + carboplatin (striped bars). Dose responses of Ishikawa cells exposed to (D, E) PFOA or (H, I) PFHpA then treated with carboplatin only (gray bars), BPD‐PDP (*hν*: 0.01 J/cm^2^) + carboplatin (checkered bars), or ALA‐PpIX‐PDP (*hν*: 0.1 J/cm^2^) + carboplatin (striped bars). Gray bars represent previously published data that has been normalized and presented in a different format. Data shown are normalized to their own no treatment control and are from *n* = at least four independent experiments with two technical replicates each. Significant differences between combination therapy‐treated exposure groups and exposure groups treated only with carboplatin are denoted by * (*p* < 0.05) and were determined using multiple unpaired *t*‐tests. Contains Servier Medical Art stock images provided by Servier, licensed under a Creative Commons Attribution 3.0 unported license.

In addition to evaluating the efficacy of PDP in overcoming PFAS‐induced platinum resistance (for select chemotherapy doses), combination therapy was also evaluated over a broader range of chemotherapy doses in HEC‐1B and Ishikawa cells. In Figure 4, dose–responses are shown for PFAS‐exposed endometrial cancer cells treated with either combination PDP + carboplatin (red or green bars) or carboplatin only (gray bars).

#### BPD‐PDP

In HEC‐1B cells, a significant reduction in survival fraction is observed in several groups exposed to PFAS followed by treatment with the combination therapy compared to the respective chemotherapy only groups (50 μM carboplatin): 2 μM PFOA (chemo only: 0.94 ± 0.08, combination: 0.73 ± 0.16, *p* < 0.001), 0.5 μM PFHpA (chemo only: 0.92 ± 0.05, combination: 0.75 ± 0.15, *p* = 0.001), and 2 μM PFHpA (chemo only: 0.92 ± 0.08, combination: 0.73 ± 0.14, *p* < 0.001; Figure 4C,F,G). Similar effects were observed for 0.5 and 2 μM PFPA, where survival fraction significantly decreased in the combination BPD‐PDP + 50 μM carboplatin treatment group compared to carboplatin only (Figure S6A,B). However, no significant reduction in survival fraction was observed when BPD‐PDP was combined with higher carboplatin doses. In fact, in PFPA‐exposed cells, survival fraction increased significantly with combination BPD‐PDP + 200 μM carboplatin treatment compared to the respective carboplatin only group, suggesting that the combination therapy is most effective with lower doses of chemotherapy in the endometrial cancer cell lines evaluated here.

**FIGURE 5:**
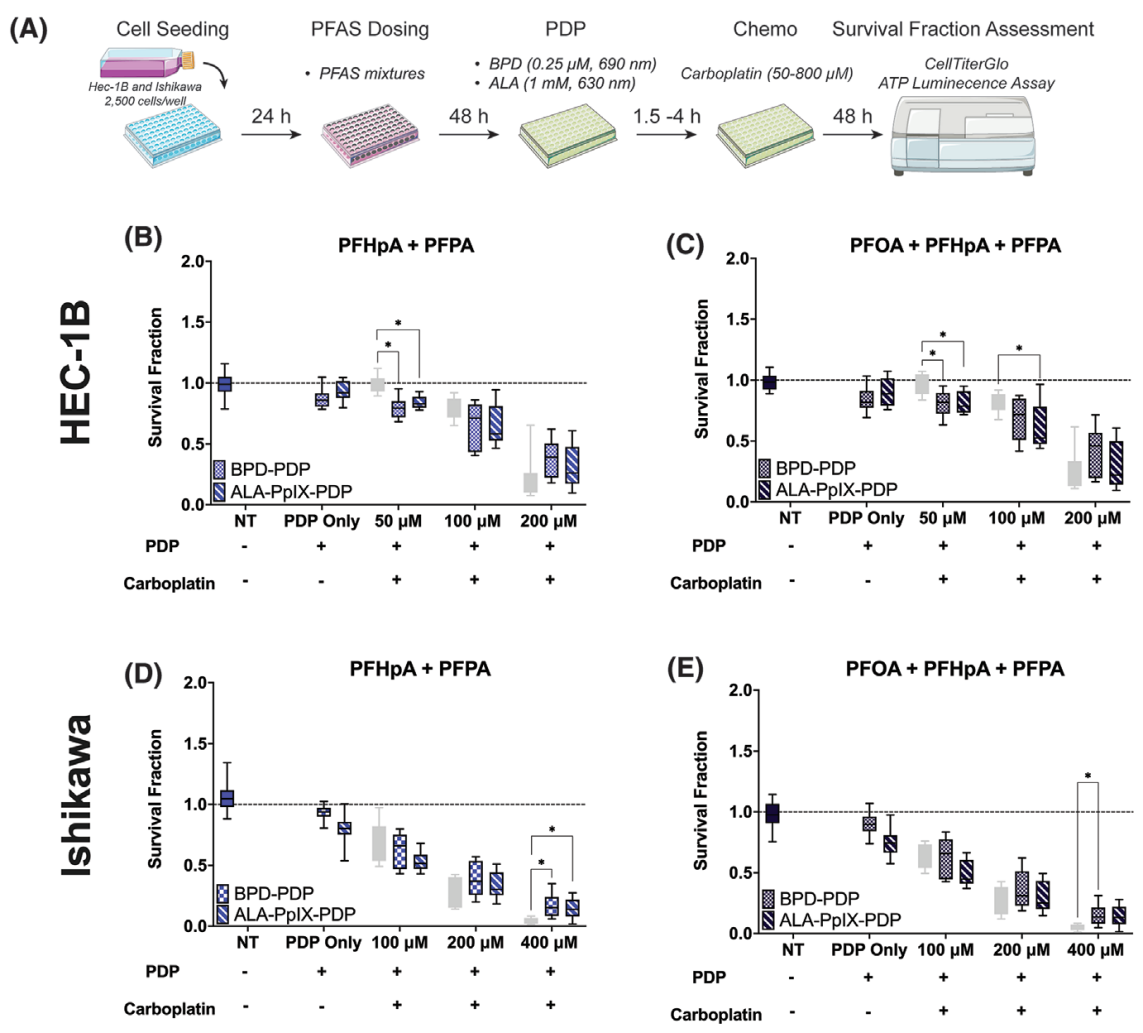
Comparison of survival fraction in HEC‐1B and Ishikawa cells exposed to PFAS mixtures then treated with carboplatin only versus combination therapy. (A) Timeline of experiments. Dose responses of HEC‐1B cells exposed to (B) PFHpA + PFPA or (C) PFOA + PFHpA + PFPA then treated with carboplatin only (gray bars), BPD‐PDP (*hν*: 0.03 J/cm^2^) + carboplatin (checkered bars), or ALA‐PpIX‐PDP (*hν*: 0.1 J/cm^2^) + carboplatin (striped bars). Dose responses of Ishikawa cells exposed to (D) PFHpA + PFPA or (E) PFOA + PFHpA + PFPA then treated with carboplatin only (gray bars), BPD‐PDP (*hν*: 0.01 J/cm^2^) + carboplatin (checkered bars), or ALA‐PpIX‐PDP (*hν*: 0.1 J/cm^2^) + carboplatin (striped bars). Gray bars represent previously published data presented in a different format. Data shown are normalized to their own no treatment control and are from *n* = at least five independent experiments with two technical replicates each. Significant differences between combination therapy‐treated exposure groups and exposure groups treated only with carboplatin are denoted by * (*p* < 0.05) and were determined using multiple unpaired *t*‐tests. Contains Servier Medical Art stock images provided by Servier, licensed under a Creative Commons Attribution 3.0 unported license.

Similar results were observed following exposure to PFAS mixtures on combination therapy response in HEC‐1B cells. Survival fraction decreased significantly only for lower doses of carboplatin, among those tested in the present study. Combination of BPD‐PDP + 50 μM carboplatin significantly reduced survival fraction for all the mixtures tested (PFHpA + PFPA, PFOA + PFHpA + PFPA, PFOA + PFHpA, PFOA + PFPA) compared to the respective carboplatin only groups (Figures 5B,C and S7A,B). The most pronounced reduction in survival fraction was observed in PFHpA + PFPA‐exposed cells following treatment with combination BPD‐PDP + 50 μM carboplatin (0.80 ± 0.09) compared to 50 μM carboplatin alone (0.99 ± 0.07, *p* < 0.001).

In Ishikawa cells, where no platinum resistance was observed following PFAS exposure,^9^ no significant reduction in survival fraction was observed with combination therapy relative to the respective chemotherapy only groups (Figure 4D,E,H,I). Similar findings were observed in Ishikawa cells exposed to PFPA or PFAS mixtures (Figures 5D,E, S6C,D and S7C,D).

#### ALA‐PpIX‐PDP

Compared to BPD‐PDP, similar results were observed when ALA‐PpIX‐PDP was used in combination with carboplatin in HEC‐1B cells. A significant decrease in survival fraction was observed in PFAS‐exposed cells treated with ALA‐PpIX‐PDP + 50 μM carboplatin compared to 50 μM carboplatin only. This occurred in HEC‐1B cells that were exposed to 2 μM PFOA (chemo only: 0.94 ± 0.08, combination: 0.81 ± 0.08, *p* = 0.003; Figure 4C) and 0.5 μM PFPA (chemo only: 0.91 ± 0.06, combination: 0.83 ± 0.03, *p* < 0.001; Figure S6A). Significant reductions in survival fraction were also observed in HEC‐1B cells exposed to PFAS mixtures then treated with combination ALA‐PpIX‐PDP + carboplatin compared to chemotherapy only controls (Figures 5B,C and S7A,B): PFHpA + PFPA (chemo only, 50 μM): 0.99 ± 0.07, combination: 0.84 ± 0.05, *p* < 0.001; PFOA + PFHpA + PFPA‐exposed cells (chemo only, 50 μM): 0.97 ± 0.08, combination: 0.82 ± 0.09; PFOA + PFHpA + PFPA (chemo only, 100 μM): 0.81 ± 0.08, combination: 0.62 ± 0.19, *p* = 0.001. Decreases in survival fraction were also observed in cells exposed to PFOA + PFHpA and PFOA + PFPA then treated with 50 μM carboplatin (Figure S7A,B). While these results suggest that in HEC‐1B cells, ALA‐PpIX‐PDP may be more effective than BPD‐PDP at combining with carboplatin to reduce survival fraction in endometrial cancer cells exposed to select PFAS or PFAS mixtures, no significant differences in photosensitizer potency were observed (Figure S8). Importantly, potency was only evaluated for PDP in combination with 100 μM carboplatin. Thus, it is possible that photosensitizer potency significantly differs when combined with lower versus higher doses of carboplatin.

In Ishikawa cells, no significant decreases in survival fraction were observed in groups that were exposed to any individual PFAS (Figures 4D,E,H,I and S6C,D), or most mixtures (Figures 5D,E and S7D) then treated with combination therapy compared to chemotherapy alone. Cells that were exposed to a mixture of PFOA + PFHpA and treated with ALA‐PpIX‐PDP in combination with 100 μM carboplatin (0.53 ± 0.06) had significantly decreased survival fraction compared to chemotherapy only (0.72 ± 0.13, *p* < 0.001; Figure S7C). Similar to Ishikawa cells treated with BPD‐PDP, several instances of increased survival fraction were observed in cells exposed to PFAS mixtures then treated with ALA‐PpIX‐PDP and 400 μM carboplatin (Figures 5D,E and S7C,D). This suggests that ALA‐PpIX‐PDP best decreases survival in combination with lower doses of chemotherapy. Similar to HEC‐1B cells, no significant differences in photosensitizer potency were observed in Ishikawa cells in any PFAS exposure group (Figure S9). Alternate ways of representing the data (normalized to their respective vehicle control at each carboplatin dose tested) displayed in Figures 3, 4, 5, S6 and S7 are presented in Figures S10–S13.

### Combination therapy using BPD‐PDP or ALA‐PpIX‐PDP and carboplatin reduces mitochondrial membrane potential (ΔΨ_m_ ) in HEC‐1B and Ishikawa cells

Although precise mechanisms underlying PFAS‐induced effects on chemotherapy resistance in endometrial cancer cells are unknown, our previous study showed that ΔΨ_m_ significantly increased in HEC‐1B and Ishikawa cells after exposure to PFAS or PFAS mixtures.^9^ Since this study focused on the use of photosensitizers for which mitochondria are either a preferential site of localization (BPD) or synthesis (ALA‐PpIX), the impact of PDP on ΔΨ_m_ (+/− chemotherapy) in PFAS‐exposed endometrial cancer cells was explored.

#### BPD‐PDP

**FIGURE 6:**
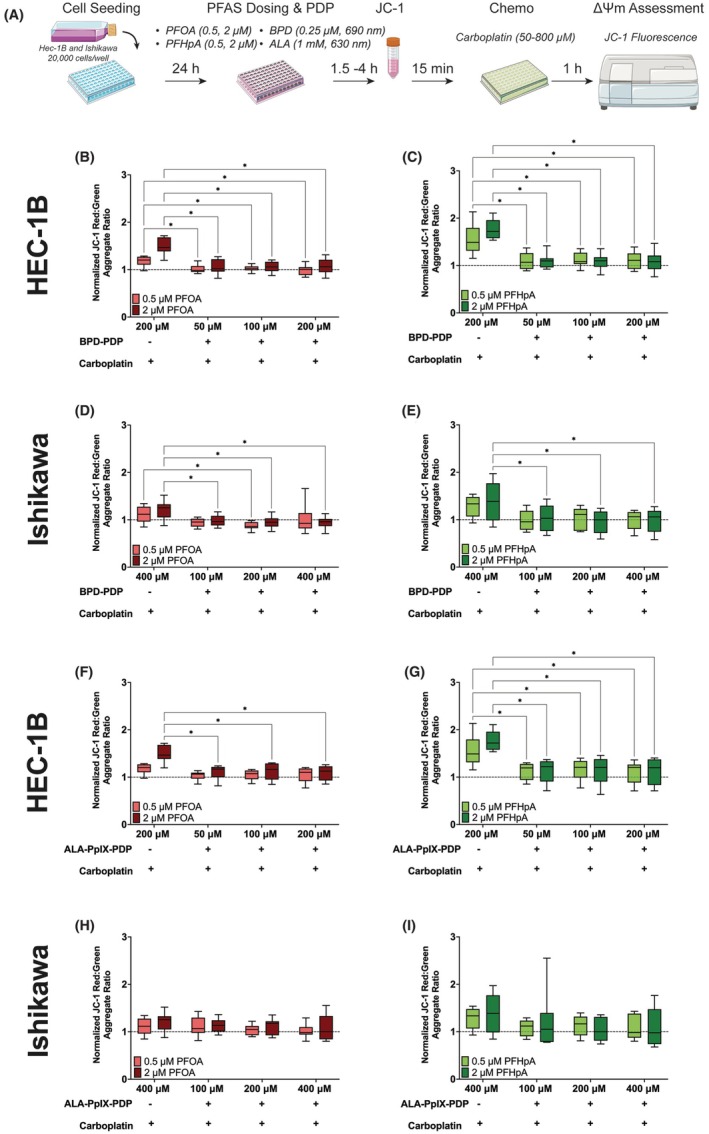
Impact of combination therapy using either BPD‐PDP (B–E) or ALA‐PpIX‐PDP (F–I) on ΔΨ_m_ in PFAS‐exposed HEC‐1B and Ishikawa cells. (A) Timeline of experiments. Comparison of ΔΨ_m_ in HEC‐1B cells that have been exposed to (B, F) PFOA or (C, G) PFHpA then treated with 200 μM carboplatin and cells treated with BPD‐PDP (*hν*: 0.03 J/cm^2^) or ALA‐PpIX‐PDP (*hν*: 0.1 J/cm^2^) in combination with carboplatin. Comparison of ΔΨ_m_ in Ishikawa cells that have been exposed to (D, H) PFOA or (E, I) PFHpA then treated with 400 μM carboplatin and cells treated with BPD‐PDP (*hν*: 0.01 J/cm^2^) or ALA‐PpIX‐PDP (*hν*: 0.1 J/cm^2^) in combination with carboplatin. Bars showing PFAS exposure group treated with carboplatin only represent previously published data.^9^ Data shown are normalized to their own vehicle control and are from *n* = at least three independent experiments with two technical replicates each. Significant differences between combination therapy‐treated exposure groups and exposure groups treated only with carboplatin are denoted by * (*p* < 0.05) and were determined using a two‐way ANOVA with Dunnett's test for multiple comparisons. Contains Servier Medical Art stock images provided by Servier, licensed under a Creative Commons Attribution 3.0 unported license.

As shown previously,^9^ ΔΨ_m_ increased in HEC‐1B cells exposed to PFOA and PFHpA, but not PFPA, then treated with carboplatin only. In the present study, ΔΨ_m_ decreased significantly in HEC‐1B cells exposed to PFOA and PFHpA at all carboplatin concentrations. Specifically, in HEC‐1B cells exposed to 2 μM PFOA then treated with BPD‐PDP + 200 μM carboplatin (JC‐1 aggregate ratio = 1.06 ± 0.15), ΔΨ_m_ decreased compared to 200 μM carboplatin‐treated only (JC‐1 aggregate ratio = 1.49 ± 0.19, *p* = 0.005; Figure 6B). A greater decrease was observed for HEC‐1B cells exposed to 2 μM PFHpA then treated with 200 μM carboplatin (JC‐1 aggregate ratio = 1.76 ± 0.21) versus those treated with BPD‐PDP + 200 μM carboplatin (JC‐1 aggregate ratio = 1.09 ± 0.22, *p* < 0.001; Figure 6C). Compared to PFOA and PFHpA, no significant changes in ΔΨ_m_ were observed for HEC‐1B cells exposed to PFPA then treated with BPD‐PDP + carboplatin (Figure S14A); however, this was expected since ΔΨ_m_ did not increase after PFPA exposure followed by carboplatin treatment alone.

Compared to HEC‐1B cells, Ishikawa cells displayed fewer instances of increased ΔΨ_m_ post‐PFAS or PFAS mixture exposure.^9^ After BPD‐PDP + carboplatin treatment, ΔΨ_m_ significantly decreased in Ishikawa cells exposed to 0.5 μM PFOA, 2 μM PFOA, and 2 μM PFHpA at nearly all carboplatin doses (Figure 6D,E). The largest decrease in ΔΨ_m_ came from cells exposed to 2 μM PFHpA (JC‐1 aggregate ratio = 1.39 ± 0.41) then treated with BPD‐PDP + 200 μM carboplatin (JC‐1 aggregate ratio = 0.96 ± 0.24, *p* = 0.005). ΔΨ_m_ was not significantly affected by combination therapy in the 0.5 μM PFHpA group; however, trends toward a decrease were observed. Additionally, despite no significant changes in ΔΨ_m_ arising from PFPA exposure + 400 μM carboplatin treatment, ΔΨ_m_ decreased significantly following combination therapy (Figure S14C).

**FIGURE 7:**
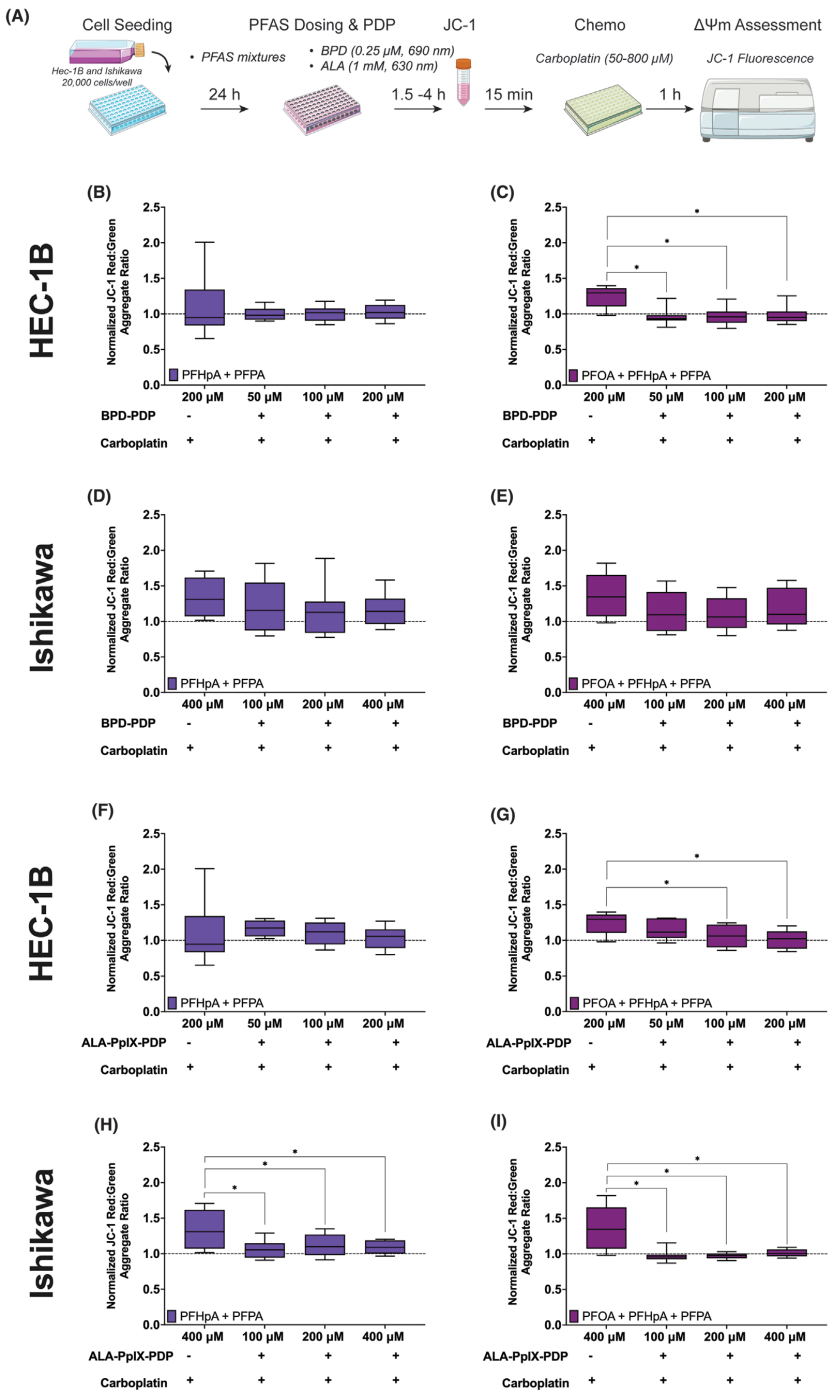
Impact of combination therapy using either BPD‐PDP (B–E) or ALA‐PpIX‐PDP (F–I) on ΔΨ_m_ in PFAS mixture‐exposed HEC‐1B and Ishikawa cells. (A) Timeline of experiments. Comparison of ΔΨ_m_ in HEC‐1B cells that have been exposed to (B, F) PFHpA + PFPA or (C, G) PFOA + PFHpA + PFPA then treated with 200 μM carboplatin and cells treated with BPD‐PDP (*hν*: 0.03 J/cm^2^) or ALA‐PpIX‐PDP (*hν*: 0.1 J/cm^2^) in combination with carboplatin. Comparison of ΔΨ_m_ in Ishikawa cells that have been exposed to (D, H) PFHpA + PFPA or (E, I) PFOA + PFHpA + PFPA then treated with 400 μM carboplatin and cells treated with BPD‐PDP (*hν*: 0.01 J/cm^2^) or ALA‐PpIX‐PDP (*hν*: 0.1 J/cm^2^) in combination with carboplatin. Bars showing PFAS exposure group treated with carboplatin only represent previously published data.^9^ Data shown are normalized to their own vehicle control and are from *n* = at least four independent experiments with two technical replicates each. Significant differences between combination therapy‐treated exposure groups and exposure groups treated only with carboplatin are denoted by * (*p* < 0.05) and were determined using a two‐way ANOVA with Dunnett's test for multiple comparisons. Contains Servier Medical Art stock images provided by Servier, licensed under a Creative Commons Attribution 3.0 unported license.

When evaluating the effects of PFAS mixtures and carboplatin treatment on ΔΨ_m_, significant increases were only observed in the PFHpA + PFPA and/or PFOA + PFHpA + PFPA for HEC‐1B and Ishikawa cells. In HEC‐1B cells exposed to PFOA + PFHpA + PFPA then treated with combination therapy, ΔΨ_m_ was decreased at all carboplatin doses (chemo only: 1.25 ± 0.15, BPD‐PDP + 50 μM carboplatin: 0.97 ± 0.12, Figure 7C), while no decreases were observed in any other mixtures exposure group (Figures 7B and S15A,B). For Ishikawa cells, no significant decreases in ΔΨ_m_ were observed for any mixtures exposure group (Figures 7D,E and S15C,D), which was unexpected as significant increases were observed after PFHpA + PFPA and PFOA + PFHpA + PFPA exposure with carboplatin treatment alone. This finding suggests that combination therapy using BPD‐PDP is not as effective at lowering ΔΨ_m_ in endometrial cancer cells exposed to PFAS mixtures compared to individual PFAS. This may also be due to PFAS mixtures affecting endometrial cancer cells via different mechanisms compared to individual PFAS.

Importantly, while combination therapy using BPD‐PDP and carboplatin was effective at lowering ΔΨ_m_ in many exposure groups across both cell lines, BPD‐PDP alone significantly lowered ΔΨ_m_ in HEC‐1B cells exposed to PFOA, PFHpA, and PFOA + PFHpA + PFPA (Figure S16). Significant decreases in ΔΨ_m_ were also observed in Ishikawa cells exposed to PFOA, PFHpA, PFPA, and PFOA + PFHpA + PFPA then treated with BPD‐PDP alone (Figure S17).

#### ALA‐PpIX‐PDP

Compared to BPD‐PDP, ALA‐PpIX‐PDP was less effective at decreasing ΔΨ_m_ in PFAS‐exposed endometrial cancer cells. This is evidenced by fewer significant differences in ΔΨ_m_ following combination therapy with ALA‐PpIX‐PDP compared to carboplatin only treated controls. ALA‐PpIX‐PDP was able to significantly decrease ΔΨ_m_ in HEC‐1B cell groups exposed to 2 μM PFOA, 0.5 μM PFHpA, and 2 μM PFHpA (Figure 6F,G). While ΔΨ_m_ was decreased to comparable levels at all carboplatin concentrations for 2 μM PFHpA, the most significant decrease occurred when cells were treated with combination therapy using 200 μM carboplatin (chemo only = 1.76 ± 0.21, combination therapy: 1.13 ± 0.27, *p* < 0.001). No changes were observed in HEC‐1B cells exposed to PFPA (Figure S14B).

ALA‐PpIX‐PDP was less effective in Ishikawa cells, which displayed moderate lowering of ΔΨ_m_ when treated with combination therapy using BPD‐PDP. No significant decreases in ΔΨ_m_ were observed for Ishikawa cells exposed to PFOA or PFHpA then treated with combination therapy using ALA‐PpIX‐PDP (Figure 6H,I). In cells exposed to 2 μM PFPA, however, significant decreases in ΔΨ_m_ were observed at all carboplatin concentrations (Figure S14D). The most significant decrease was observed when cells were treated with ALA‐PpIX‐PDP + 100 μM carboplatin (JC‐1 aggregate ratio = 0.99 ± 0.16) compared to chemotherapy only controls (JC‐1 aggregate ratio = 1.27 ± 0.31, *p* = 0.001).

When examining the effects of combination therapy using ALA‐PpIX‐PDP on endometrial cancer cells exposed to PFAS mixtures, no significant changes in ΔΨ_m_ were observed in either cell line when exposed to PFOA + PFHpA or PFOA + PFPA (Figure S15E–H). Decreases in ΔΨ_m_ were observed for HEC‐1B cells exposed to PFOA + PFHpA + PFPA and Ishikawa cells exposed to PFHpA + PFPA or PFOA + PFHpA + PFPA then treated with combination therapy (Figure 7F–I). For HEC‐1B cells, the largest decrease was observed when ALA‐PpIX‐PDP was combined with 200 μM carboplatin, while the largest decreases for Ishikawa cells were observed when ALA‐PpIX‐PDP was combined with 100 μM carboplatin (lowest dose). Again, it is important to note that, in some cases, ALA‐PpIX‐PDP alone significantly ΔΨ_m_ decreased in HEC‐1B (Figure S16; PFOA and PFHpA) and Ishikawa cells (Figure S17; PFHpA, PFPA, PFOA + PFHpA + PFPA), highlighting the value of PDP for targeting mitochondrial enhancements induced by PFAS exposure. Altogether, regardless of photosensitizer used, in most cases where ΔΨ_m_ was lowered, it was reduced to comparable levels at all carboplatin doses. This indicates that PDP in combination with low‐dose chemotherapy is just as effective as PDP in combination with higher doses of chemotherapy at lowering ΔΨ_m_.

## DISCUSSION

Environmental contamination by PFAS is a global concern, especially considering that these chemicals are associated with a variety of adverse health outcomes.^21^ In the context of women's health, PFAS have been linked with disorders like endometriosis, which can increase the risk of developing endometrial cancer later in life.^23^, ^24^, ^25^ PFAS are also associated with obesity and dysregulation of menstruation and hormones, which can also impact fertility status and cancer risk.^22^, ^41^, ^42^, ^43^ Prior to our recently published studies,^9^, ^19^ the effects of PFAS exposure on chemotherapy response in any cancer had never been examined. This is problematic, as humans are likely exposed to these chemicals daily through a variety of routes (drinking water, food supplies, dental floss, cosmetics, food packaging, etc.).^27^, ^44^ In fact, since the main route of exposure is drinking water,^44^, ^45^, ^46^, ^47^ the U.S. Environmental Protection Agency (EPA) recently implemented the first piece of legislation restricting the legal amount of PFAS in drinking water.^48^ While this represents a major step forward, this legislation would only regulate levels of six individual PFAS chemicals out of an estimated total of ~14,000. Nonetheless, this effort to mitigate environmental contamination of PFAS is critical, as it is estimated that over 200 million Americans are exposed to PFAS at levels higher than what's been determined as safe by the National Primary Drinking Water Regulations.^49^

Due to their chemically inert nature and lipid‐mimicking physiochemical properties, PFAS are bioaccumulative with long half‐lives (on the order of months or years). Thus, it is plausible that after decades of PFAS exposure, a person's ability to respond to chemotherapy may be negatively impacted. Both ovarian cancer and endometrial cancer cell lines demonstrate platinum resistance after just 48 h of PFAS exposure.^9^, ^19^ Importantly, depending on which cancer cell type was evaluated, PFAS and PFAS mixtures that induced platinum resistance differed. In endometrial cancer cells, platinum resistance was observed after exposure to PFOA, PFHpA, and PFPA, but not PFAS mixtures. Conversely, ovarian cancer cells displayed platinum resistance after exposure to PFHpA, PFPA, and PFAS mixtures, but not PFOA alone. These findings demonstrate the ability of several PFAS to impact chemotherapy response in a cancer‐specific fashion.

Since chemoresistance is responsible for an estimated 90% of deaths in patients with advanced‐stage gynecologic malignancies,^50^, ^51^, ^52^ the present study evaluated the ability of PDP, a non‐mechanistically overlapping therapeutic approach, in combination with low‐dose carboplatin to overcome PFAS‐induced platinum resistance in endometrial cancer cells. The use of two clinically‐relevant, FDA‐approved photosensitizers shown to induce mitochondrial photodamage (BPD and ALA‐PpIX) was compared.^53^, ^54^, ^55^ Diminishing mitochondrial health is of interest in PFAS‐exposed cells since ΔΨ_m_ increased in both ovarian and endometrial cancer cells following PFAS exposure and carboplatin treatment,^9^, ^19^ indicating a potential role for mitochondria in PFAS‐induced platinum resistance.

Significant reductions in survival fraction were observed in HEC‐1B and Ishikawa cells exposed to PFAS then treated with BPD‐PDT or ALA‐PpIX‐PDT compared to controls that were not exposed to PFAS. Interestingly, these decreases were only observed in HEC‐1B cells treated with BPD‐PDT and in Ishikawa cells treated with ALA‐PpIX‐PDT. This could indicate increased sensitivity to PDT following PFAS exposure; however, this effect was only observed at select light doses for each photosensitizer and only in some exposure groups depending on the cell line. Additionally, these effects were much less pronounced than those observed in ovarian cancer cells treated with PDP, where curves either trended, or significantly shifted, upward after PFAS exposure, indicating decreased sensitivity to PDT.^27^ Vehicle‐induced effects were also observed in Ishikawa cells treated with BPD‐PDT, potentially suggesting increased sensitivity of this cell line to 1.0 N potassium hydroxide in methanol. This vehicle was selected based on studies showing enhanced solubility and stability of PFAS^56^; however, we are actively searching for a less toxic means of solubilizing PFAS that does not sacrifice chemical stability.

It is also important to note that Ishikawa cells, in the absence of exposure to PFAS or vehicle, were much more sensitive to BPD‐PDT than previous reports in ovarian cancer cell lines^27^ and HEC‐1B cells in the current study. BPD‐PDT in Ishikawa cells substantially reduced survival fraction at the lowest energy density (0.03 J/cm^2^) at an irradiance of 20.12 mW/cm^2^ (irradiation time: 1 s; Figure S1). The same energy density at an irradiance of 6.71 mW/cm^2^ led to decreased toxicity (Figure 1C). Prior studies by us and others have shown irradiance‐dependent effects on PDT response in a variety of cancer cell lines.^57^, ^58^, ^59^, ^60^ These effects have been reported in the context of tumor spheroids, adherent 3D tumor models, and in vivo tumors. Among the factors that have been considered in these studies are variations in photosensitizer and light distribution, rates of photobleaching, tumor oxygenation, and molecular signaling pathways. Since the endometrial cancer cell lines in the present study were treated in monolayer cultures, biological factors such as stress response mechanisms and survival pathways, as well as the impact of varying PDT‐dose parameters in 3D cultures,^59^ will be investigated in future studies. It is important to note that low light doses and brief irradiation times were used in the present study. Differences in instrument variability could have led to small, but non‐negligible, differences in energy densities. Another possible concern is the contribution of ambient light to the observed PDT response, although standard precautions were taken to abrogate the effects of ambient lighting (lights turned off and/or plates wrapped in foil during transport), and photosensitizer only (dark) controls were included on each plate.

Since no studies have evaluated BPD toxicity in Ishikawa cells, it is also possible that the observed increase in BPD‐PDT efficacy could result from decreased expression of *p*‐glycoprotein or adenosine triphosphate (ATP)‐binding cassette G2 (ABCG2), as these are responsible for cellular efflux of BPD.^61^ It is also possible that the unique oncogenic mutational profiles of these cell lines impact mitochondrial function and therefore BPD‐PDT efficacy. HEC‐1B cells are known to harbor PIK3CA and KRAS mutations, both of which can alter mitochondrial metabolism, while Ishikawa cells are known to harbor a PTEN mutation, which may impact mitophagy regulation.^62^, ^63^, ^64^, ^65^ These unique cell‐based differences in response to PFAS and treatments may provide possible clues to varied biological markers in patients that could move the field of precision medicine.

While the irradiance and light dose range used for ALA‐PpIX‐PDT in Ishikawa cells was the same as HEC‐1B cells, increased efficacy was noted in Ishikawa cells. The IC_50_ light dose in Ishikawa cells treated with ALA‐PpIX‐PDT was 0.6 J/cm^2^ while that of HEC‐1B cells was 1.8 J/cm^2^. The observed difference in efficacy of ALA‐PpIX‐PDT between cell lines and following PFAS exposures is interesting and warrants further study involving heme biosynthesis, hormone receptor activity, and oncogenic signaling pathways.^66^, ^67^, ^68^, ^69^, ^70^, ^71^, ^72^, ^73^, ^74^, ^75^, ^76^ These evaluations are critical, as PFAS have been shown to display estrogenic activity in vitro and in vivo^77^, ^78^ and may influence the development of conditions such as polycystic ovarian syndrome and uterine fibroids.^79^, ^80^

In our recently published study^9^ evaluating PFAS‐induced platinum resistance in endometrial cancer cells, resistance was only observed in HEC‐1B cells; in fact, Ishikawa cells, in some cases, had decreased survival fraction post‐PFAS exposure and carboplatin treatment, suggestive of increased chemotherapy sensitivity. Since platinum‐resistance was observed in HEC‐1B cells, the present study evaluated the ability of BPD‐PDP or ALA‐PpIX‐PDP in combination with carboplatin to overcome platinum resistance arising from PFAS exposure. Importantly, in all groups where platinum resistance was observed, PDP lowered HEC‐1B survival fraction, albeit not significantly in all cases. More instances of significant reductions in survival fraction were observed in HEC‐1B cells treated with ALA‐PpIX‐PDP rather than BPD‐PDP, indicating increased sensitivity of PFAS‐exposed HEC‐1B cells to ALA‐PpIX in combination with 200 μM carboplatin. This is the opposite of what was observed in ovarian cancer cells, where BPD‐PDP was more potent than ALA‐PpIX‐PDP.^27^ This suggests differences in sensitivity of PFAS‐exposed cancer cells to PDP and warrants further investigation to inform clinical treatment regimens. When comparing dose responses of PFAS‐exposed endometrial cancer cells treated with carboplatin only versus PDP + carboplatin, analyses illustrated that PDP in combination with lower doses of carboplatin (50–100 μM) significantly reduced survival fractions in some cases; however, when PDP was combined with higher doses of carboplatin (200–400 μM), several instances of increased survival fraction were observed compared to carboplatin only. This indicates decreased sensitivity of PFAS‐exposed endometrial cancer cells to combination therapy when higher carboplatin doses are used and warrants further investigation. It is possible that PFAS induce platinum resistance in endometrial cancer cells via multiple pathways, and that mitochondrial pathways may only represent one avenue by which low‐dose combination therapy can overcome resistance.

Since mitochondria are targets of interest for PFAS and platinum resistance, this study evaluated the effects of combination therapy on ΔΨ_m_. Following PDP alone and combination therapy, significant decreases in ΔΨ_m_ were observed compared to PFAS‐exposed cells treated with carboplatin only. In many cases, especially those where ΔΨ_m_ increased post‐PFAS exposure or carboplatin resistance was observed, BPD‐PDP and ALA‐PpIX‐PDP in combination with carboplatin decreased ΔΨ_m_ to around 1.0. While this generally indicates that the cell is not yet undergoing apoptosis (ΔΨ_m_ < 1), this is similar to the effect of combination therapy on ovarian cancer cells^27^ and may result from insufficient assay length required to capture downstream effects (i.e., decreases observed after the 1‐h assay may continue, leading the cell towards apoptosis). While combination therapy using ALA‐PpIX‐PDP reduced survival fraction more than that of BPD‐PDP in PFAS‐induced, platinum‐resistant endometrial cancer cells, BPD‐PDP appeared more effective at reducing ΔΨ_m_. This could suggest that BPD‐PDP is better at inducing mitochondrial photodamage in endometrial cancer cells while ALA‐PpIX‐PDP works through additional mechanisms that remain to be explored. It is also possible that ALA‐PpIX is less effective at accumulating in mitochondria of PFAS‐exposed cells, leading to limited PDP‐induced mitochondrial damage.

One strength of this study is that two mitochondria‐associated photosensitizers for PDP were compared, for the first time, in two mutationally diverse endometrial cancer cell lines. These findings may provide novel insight into the potential clinical benefits of these photosensitizers, based on differences in potency and efficacy, specifically for use in patients with elevated PFAS exposures. Another strength of this study is that PFAS chemicals, mixtures, and concentrations were selected based on their relevance to contaminated communities. Despite these strengths, several limitations provide direction for future studies. While this study evaluated ΔΨ_m_ as a preliminary mechanism by which PDP overcomes PFAS‐induced platinum resistance in endometrial cancer cells, the JC‐1 dye has known limitations and more quantitative approaches to measure ΔΨ_m_ (i.e., flow cytometry) are available. Additionally, evaluation of other mitochondrial structural or functional endpoints like mitochondrial deoxyribonucleic acid (mtDNA) copy number and metabolic status may provide insight into more precise mechanisms underlying platinum resistance. Finally, while this study provides novel insight into overcoming detrimental effects of short‐term PFAS exposures, chronic PFAS exposures (weeks–months) are more human‐relevant and warrant exploration in endometrial cancer cell lines.

In conclusion, this study reports, for the first time, the ability of PDP using either BPD or ALA‐PpIX to overcome PFAS‐induced platinum resistance in endometrial cancer cell lines. Combination therapy using BPD‐PDP or ALA‐PpIX‐PDP with low‐dose carboplatin reduced not only survival fraction, but also ΔΨ_m_, suggesting a potential role for mitochondrial dysfunction in platinum resistance.

## Supporting information


Data S1.

## Data Availability

The data that support the findings of this study are available from the corresponding author upon reasonable request.
