# Supplementary material for: Photodynamic priming overcomes platinum resistance from short‐term exposure to select perfluoroalkyl substances in endometrial cancer cell lines
Source: Photochem Photobiol. 2025 Mar 10;101(5):1177–98. doi: 10.1111/php.14073 (PMC12355225; doi:10.1111/php.14073)
Supplement: Supplementary file 1 — Data S1. [file PHP-101-1177-s001.docx]

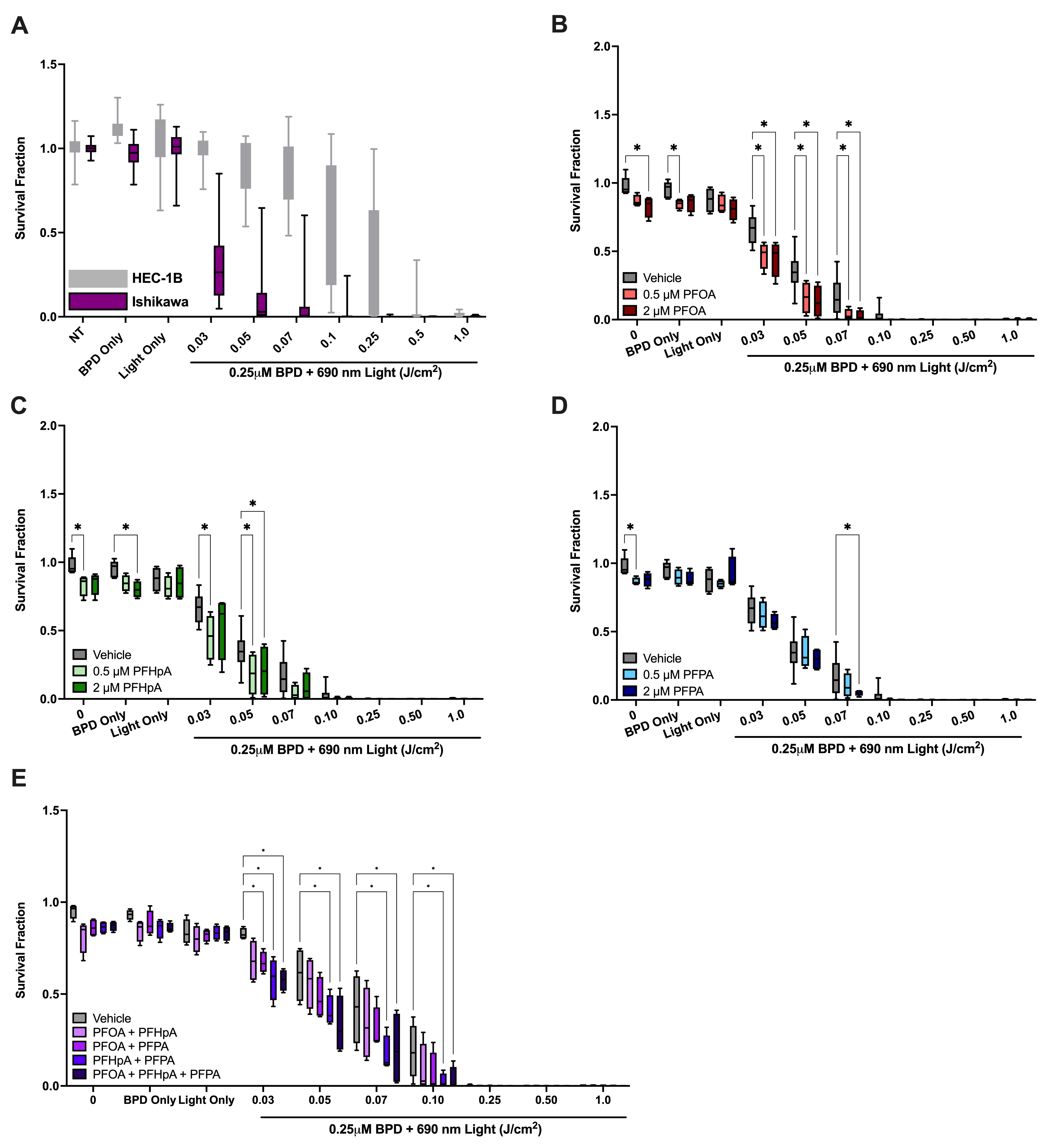


**Figure S1. Efficacy of BPD-PDT at 20.12 mW/cm^2^ in Ishikawa cells**. A) BPD-PDT dose response in Ishikawa cells when using light dose range comparable to HEC-1B cells. BPD-PDT dose response (20.12 mW/cm^2^) in Ishikawa cells exposed to B) PFOA, C) PFHpA, D) PFPA, or E) PFAS mixtures. Data shown are normalized to their own no treatment control and are from n= at least 2 independent experiments with at least two technical replicates each. Significant differences between PFPA-exposed groups and vehicle-exposed groups are denoted by * (*p* < 0.05) and were determined using a two-way ANOVA with Dunnett’s test for multiple comparisons.


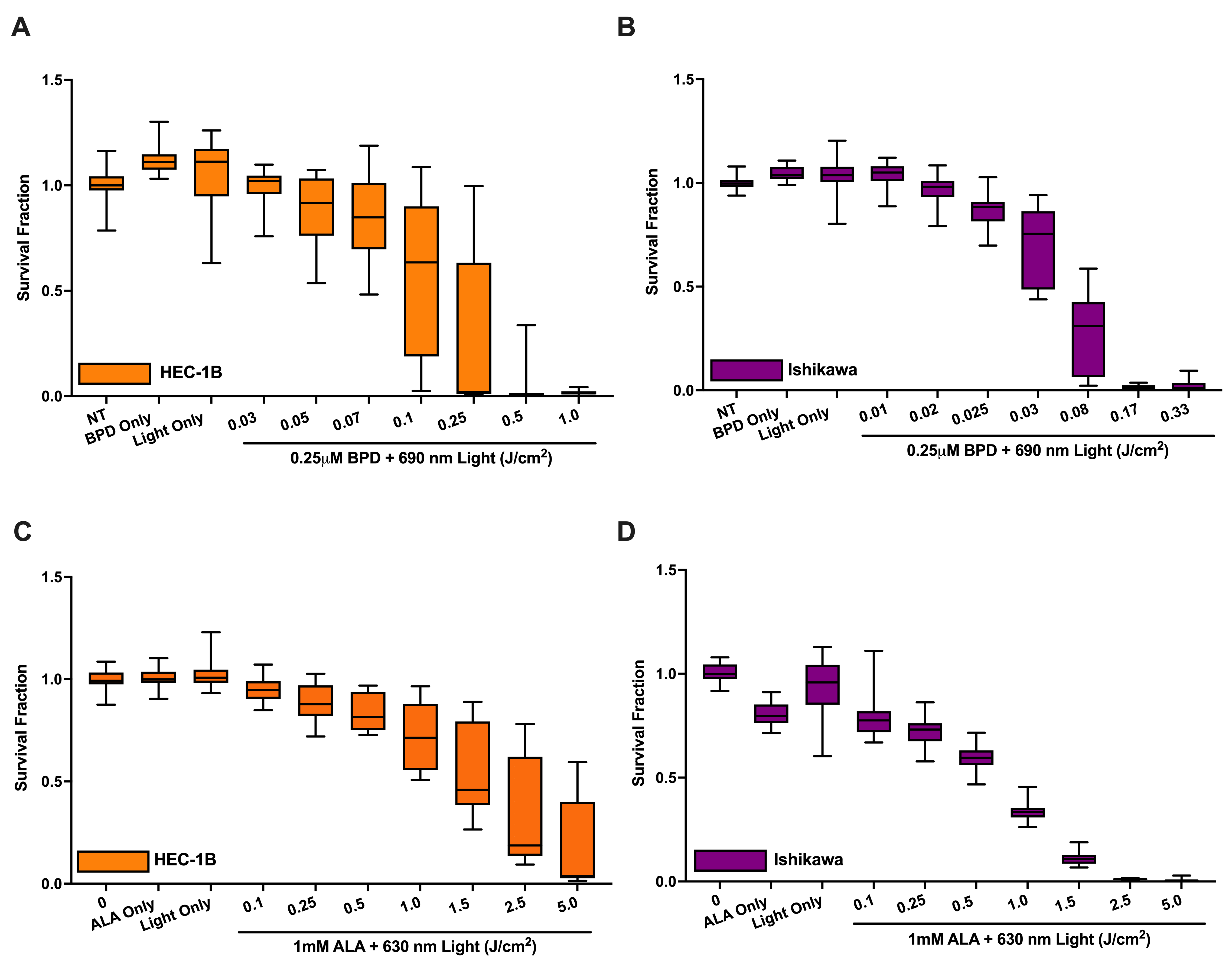


**Figure S2. Efficacy of BPD-PDT (A&B) and ALA-PpIX-PDT (C&D) in HEC-1B and Ishikawa cells**. Dose responses of BPD-PDT in A) HEC-1B and B) Ishikawa cells or ALA-PpIX-PDT in C) HEC-1B and D) Ishikawa cells. Data shown are normalized to their own no treatment control and are from n= at least 3 independent experiments with six technical replicates each.


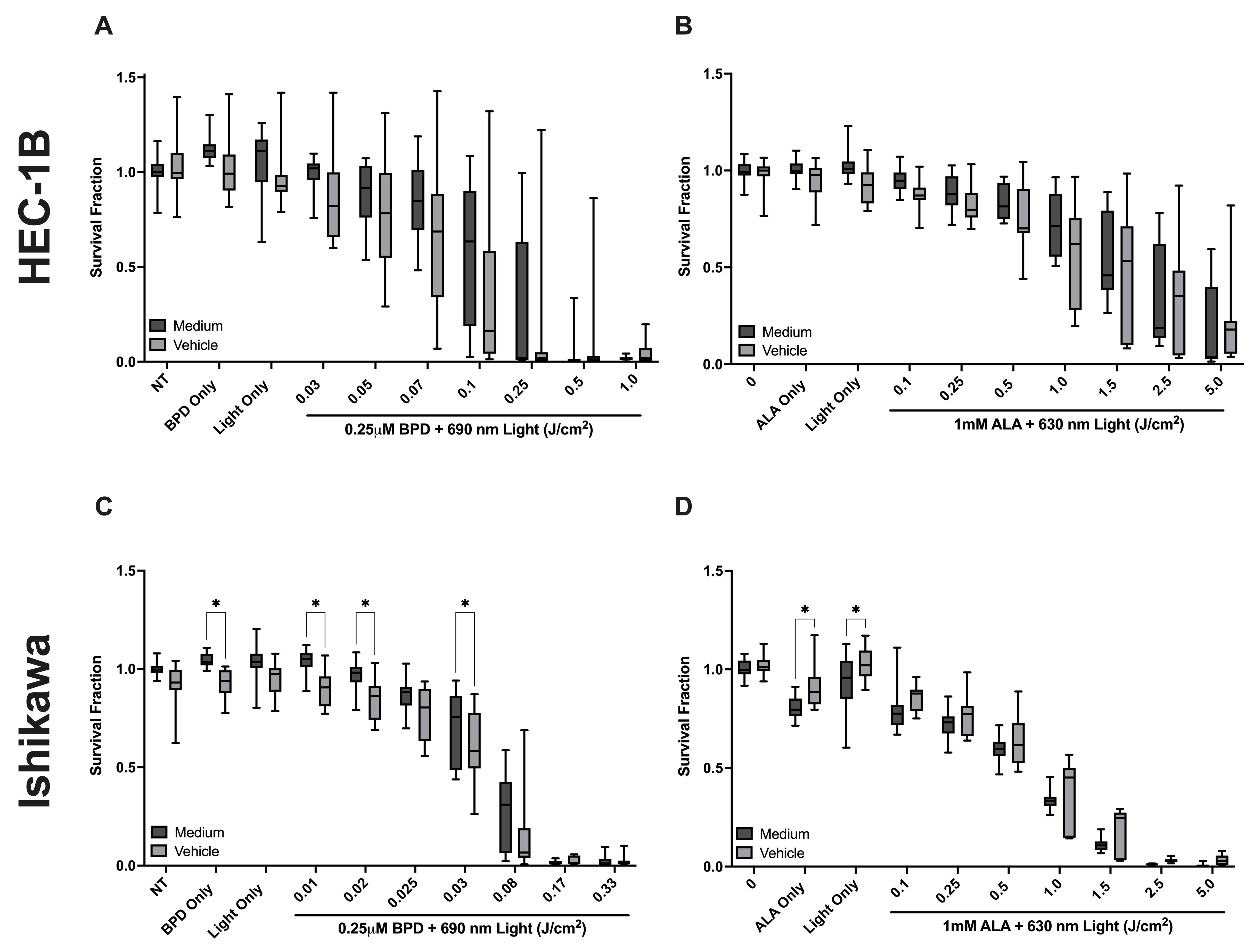


**Figure S3. Comparison of BPD-PDT (A&C) and ALA-PpIX-PDT (B&D) efficacy in unexposed versus vehicle-exposed endometrial cancer cell lines**. BPD-PDT dose responses in untreated and vehicle-exposed A) HEC-1B and C) Ishikawa cells. ALA-PpIX-PDT dose responses in untreated and vehicle-exposed B) HEC-1B and D) Ishikawa cells. Data shown are normalized to their own no treatment control and are from n= at least 4 independent experiments with at two technical replicates each. Significant differences between vehicle-exposed groups and unexposed groups are denoted by * (*p* < 0.05) and were determined using a two-way ANOVA with Dunnett’s test for multiple comparisons.


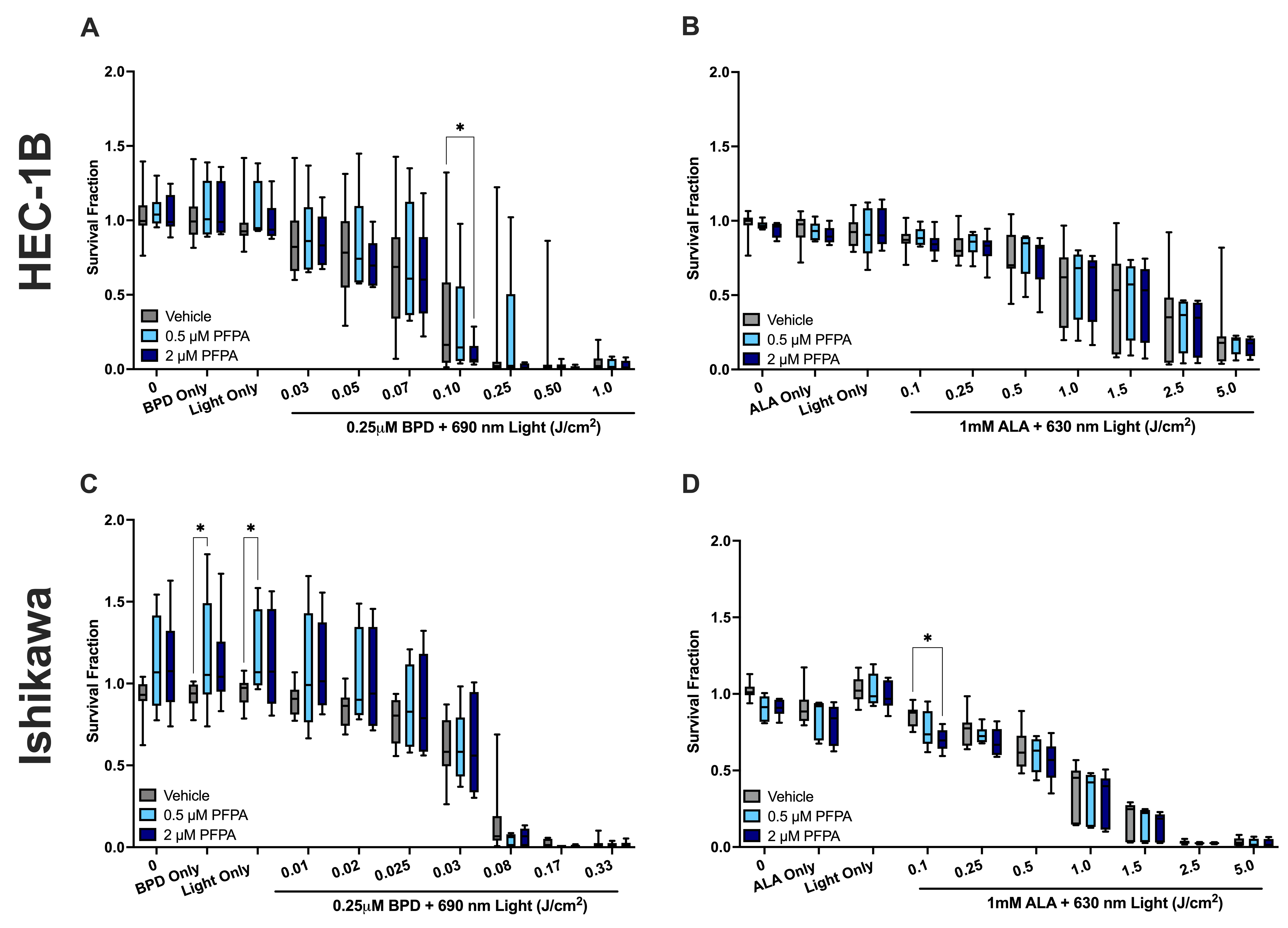


**Figure S4. Efficacy of BPD-PDT (A&C) and ALA-PpIX-PDT (B&D) in HEC-1B and Ishikawa cells exposed to PFPA**. Dose responses of BPD-PDT in A) HEC-1B cells or C) Ishikawa cells exposed to PFPA. Dose responses of ALA-PpIX-PDT in B) HEC-1B or D) Ishikawa cells exposed to PFPA. Data shown are normalized to their own no treatment control and are from n= at least 3 independent experiments with two technical replicates each. Significant differences between PFPA-exposed groups and vehicle-exposed groups are denoted by * (*p* < 0.05) and were determined using a two-way ANOVA with Dunnett’s test for multiple comparisons.


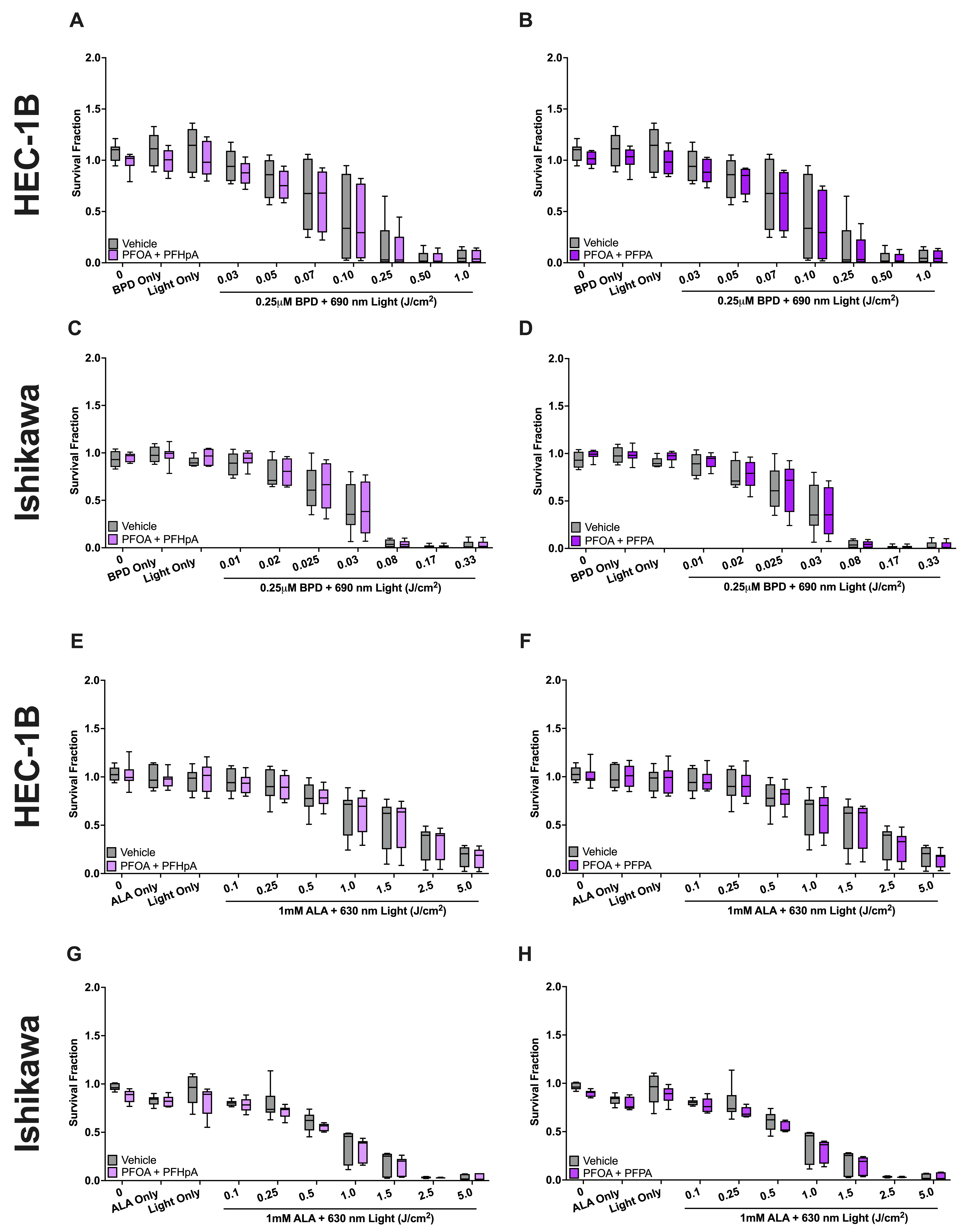


**Figure S5. Evaluation of BPD-PDT (A-D) and ALA-PpIX-PDT (E-H) in HEC-1B and Ishikawa cells exposed to PFAS mixtures**. Dose responses of BPD-PDT in HEC-1B cells exposed to A) PFOA + PFHpA and B) PFOA + PFPA or Ishikawa cells exposed to C) PFOA + PFHpA and D) PFOA + PFPA. Dose responses of ALA-PpIX-PDT in HEC-1B cells exposed to E) PFOA + PFHpA and F) PFOA + PFPA or Ishikawa cells exposed to G) PFOA + PFHpA and H) PFOA + PFPA. Data shown are normalized to their own no treatment control and are from n= at least 3 independent experiments with two technical replicates each.


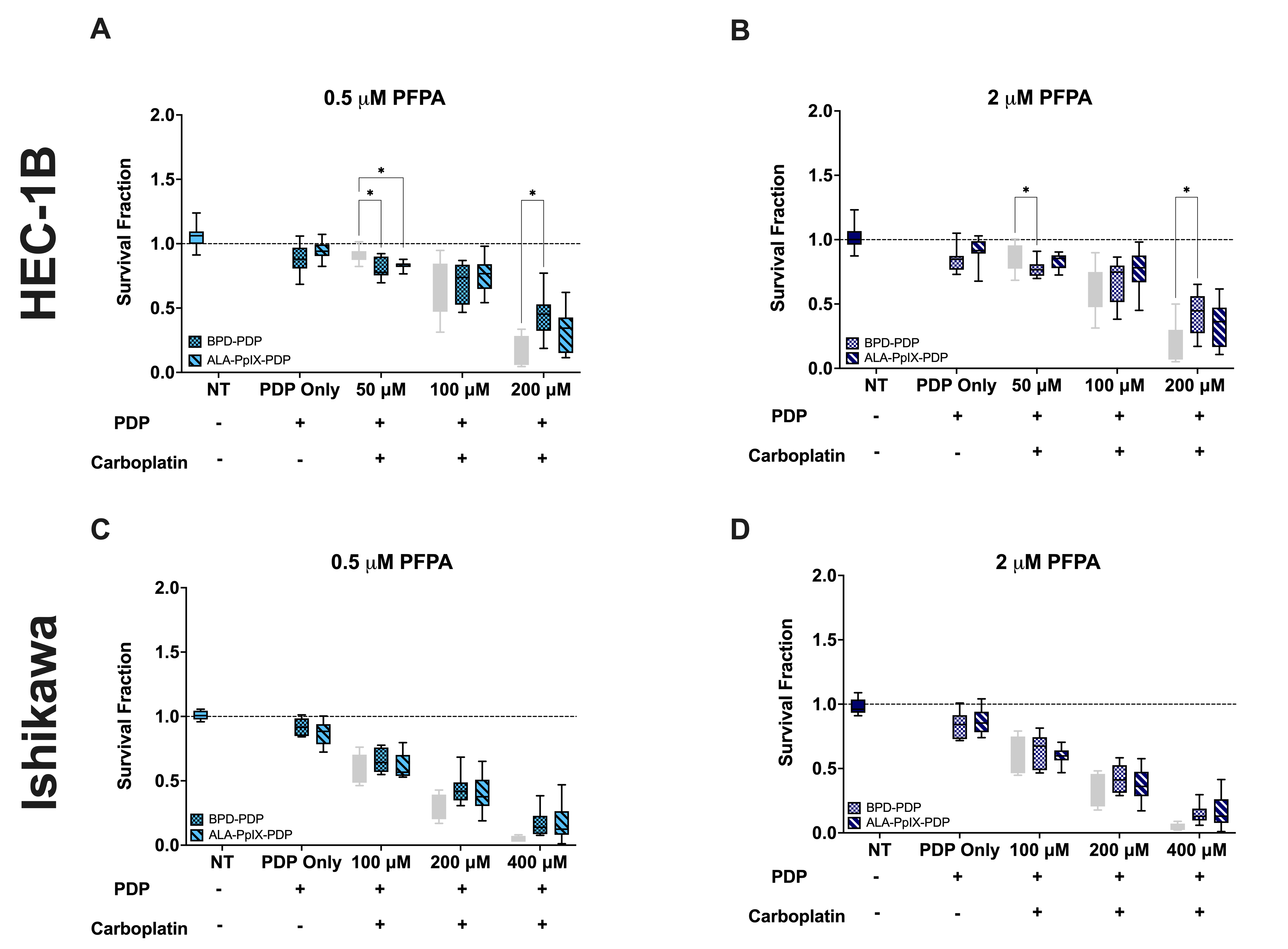


**Figure S6. Comparison of survival fraction in HEC-1B and Ishikawa cells exposed to PFPA then treated with carboplatin only versus combination therapy**. Dose responses of HEC-1B and Ishikawa cells exposed to A,C) 0.5 μM PFPA or B,D) 2 μM PFPA then treated with carboplatin only (grey bars), BPD-PDP (hν: 0.03 J/cm^2^) + carboplatin (checkered bars), or ALA-PpIX-PDP (hν: 0.1 J/cm^2^) + carboplatin (striped bars). Grey bars represent previously published data that has been normalized and presented in a different format. Data shown are normalized to their own no treatment control and are from n= at least 4 independent experiments with two technical replicates each. Significant differences between combination therapy-treated exposure groups and exposure groups treated only with carboplatin are denoted by * (*p* < 0.05) and were determined using multiple unpaired t-tests.


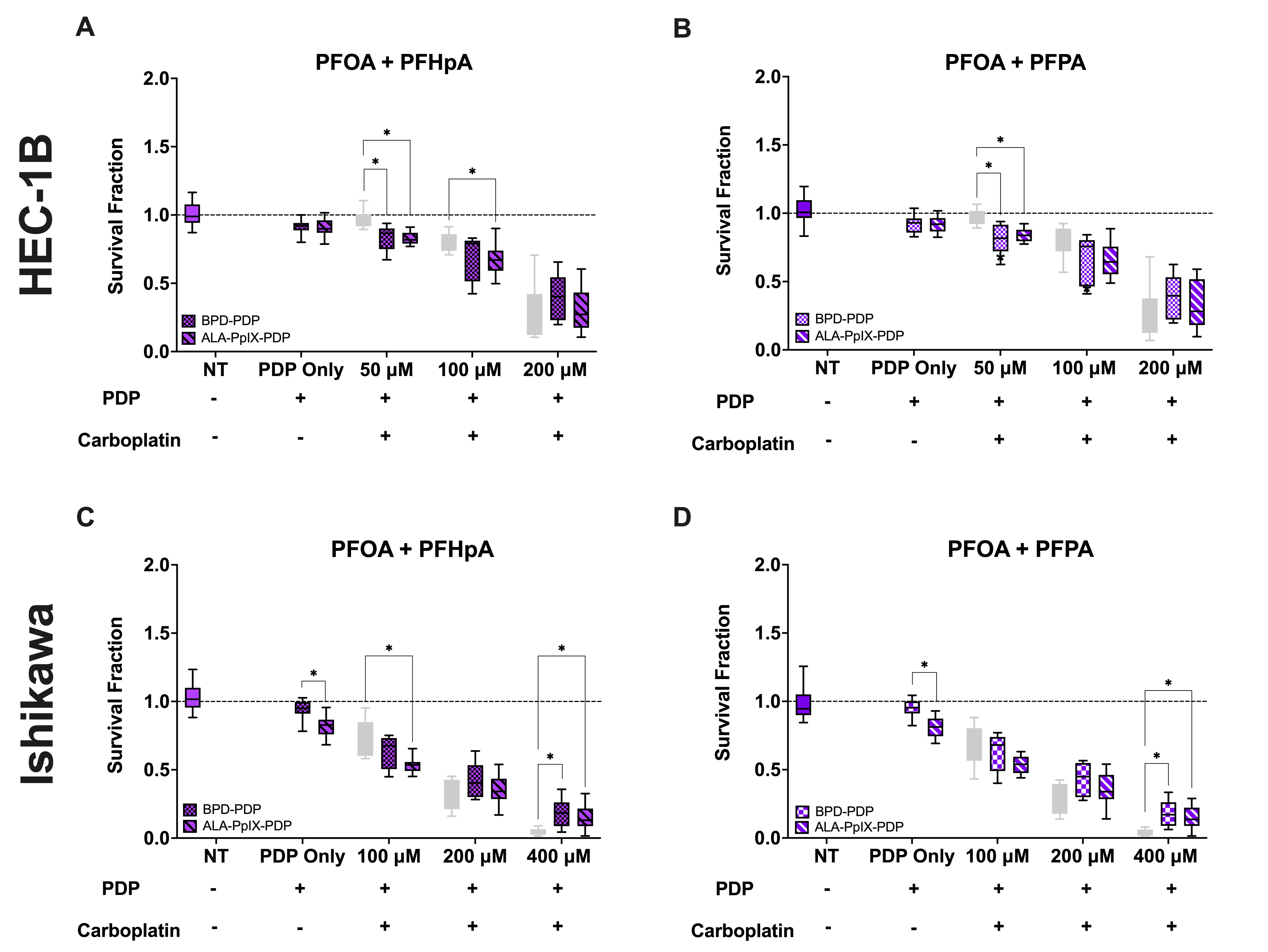


**Figure S7. Survival fraction in HEC-1B and Ishikawa cells exposed to PFAS mixtures then treated with carboplatin only versus combination therapy**. Dose responses of HEC-1B and Ishikawa cells exposed to A,C) PFOA + PFHpA or B,D) PFOA + PFPA then treated with carboplatin only (grey bars), BPD-PDP (hν: 0.03 J/cm^2^) + carboplatin (checkered bars), or ALA-PpIX-PDP (hν: 0.1 J/cm^2^) + carboplatin (striped bars). Grey bars represent previously published data that has been normalized and presented in a different format. Data shown are normalized to their own no treatment control and are from n= at least 5 independent experiments with two technical replicates each. Significant differences between combination therapy-treated exposure groups and exposure groups treated only with carboplatin are denoted by * (*p* < 0.05) and were determined using multiple unpaired t-tests.


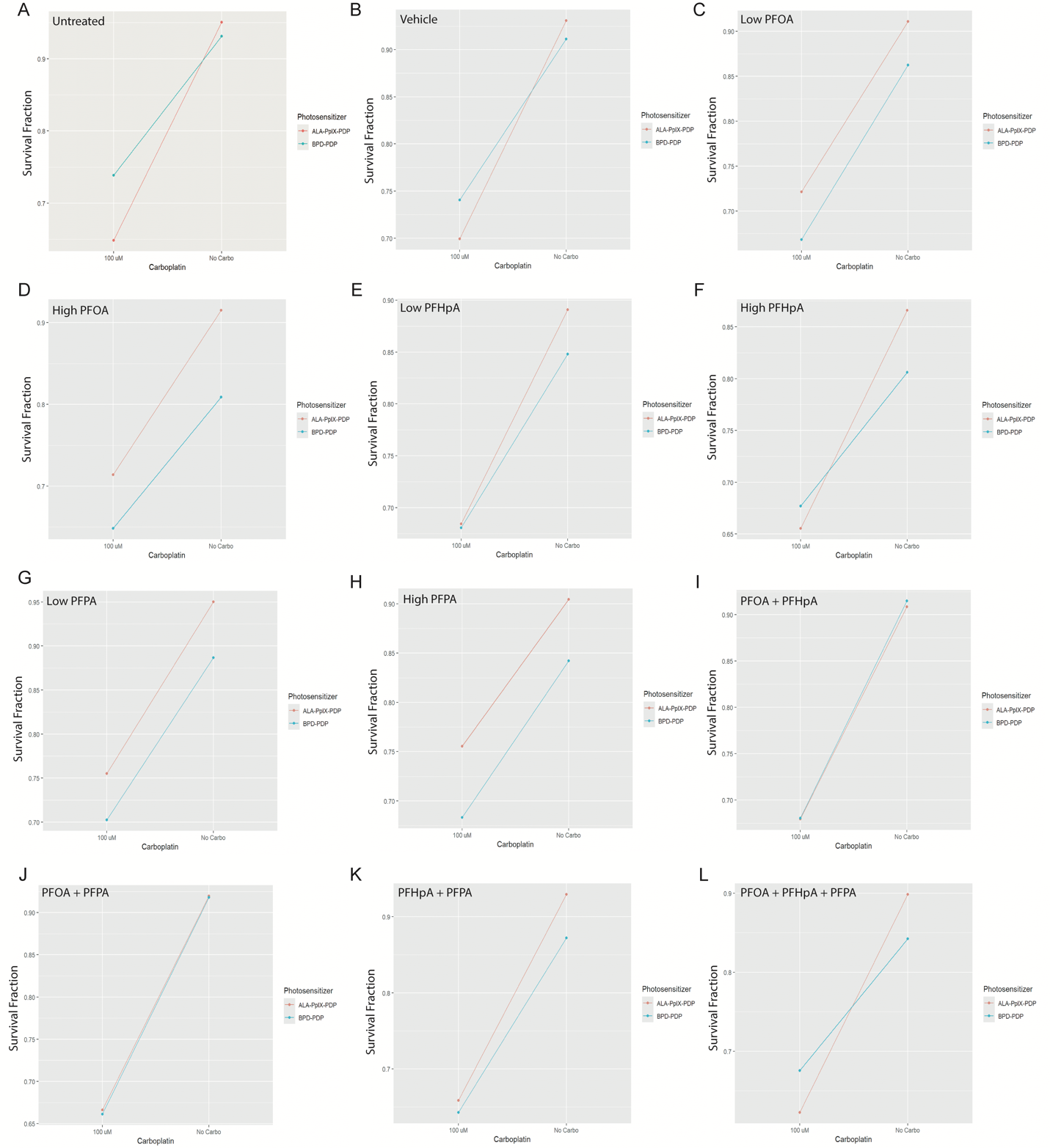


**Figure S8. In HEC-1B cells, photosensitizer efficacy for PDP in combination with 100 μM carboplatin** **did not differ between BPD and ALA-PpIX.** The interaction between BPD-PDP (hν: 0.03 J/cm^2^) + 100 μM carboplatin and ALA-PpIX-PDP (hν: 0.10 J/cm^2^) + 100 μM carboplatin was not significant in (A) untreated [*p* = 0.183], (B) vehicle- [*p* = 0.286], (C) 0.5 μM PFOA- [*p* = 0.949], (D) 2 μM PFOA- [*p* = 0.533], (E) 0.5 μM PFHpA- [*p* = 0.603], (F) 2 μM PFHpA- [*p* = 0.285], (G) 0.5 μM PFPA- [*p* = 0.857], (H) 2 μM PFPA- [*p* = 0.880], (I) PFOA + PFHpA- [*p* = 0.935], (J) PFOA + PFPA- [*p* = 0.960], (K) PFHpA + PFPA- [*p* = 0.567], and (L) PFOA + PFHpA + PFPA-exposed HEC-1B cells [*p* = 0.110].


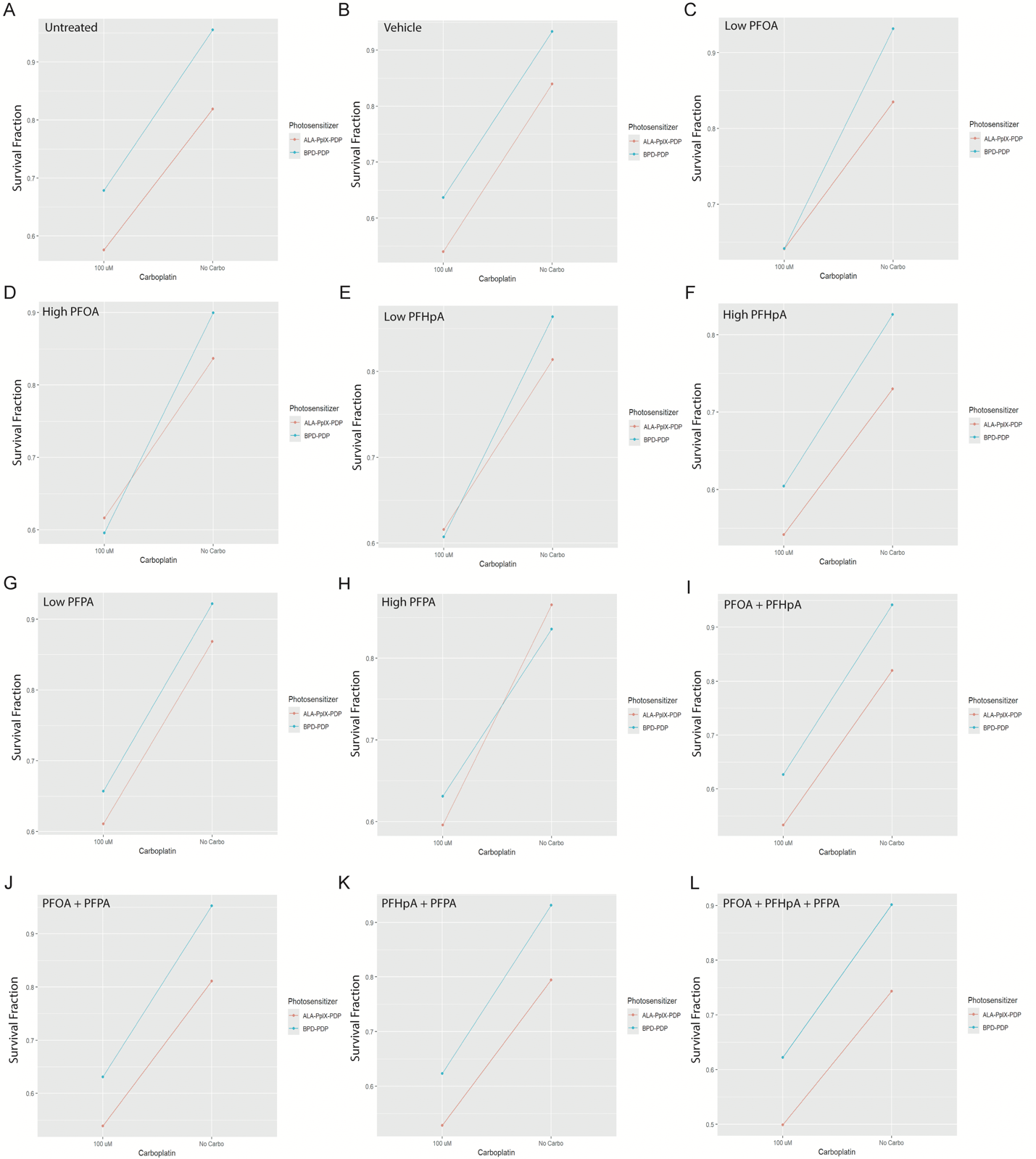
 **Figure S9. In Ishikawa cells, photosensitizer efficacy for PDP in combination with 100 μM carboplatin** **did not differ between BPD and ALA-PpIX.** The interaction between BPD-PDP (hν: 0.01 J/cm^2^) + 100 μM carboplatin and ALA-PpIX-PDP (hν: 0.10 J/cm^2^) + 100 μM carboplatin was not significant in (A) untreated [*p* = 0.474], (B) vehicle- [*p* = 0.938], (C) 0.5 μM PFOA- [*p* = 0.161], (D) 2 μM PFOA- [*p* = 0.166], (E) 0.5 μM PFHpA- [*p* = 0.348], (F) 2 μM PFHpA- [*p* = 0.586], (G) 0.5 μM PFPA- [*p* = 0.877], (H) 2 μM PFPA- [*p* = 0.315], (I) PFOA + PFHpA- [*p* = 0.567], (J) PFOA + PFPA- [*p* = 0.298], (K) PFHpA + PFPA- [*p* = 0.435], and (L) PFOA + PFHpA + PFPA-exposed Ishikawa cells [*p* = 0.514].


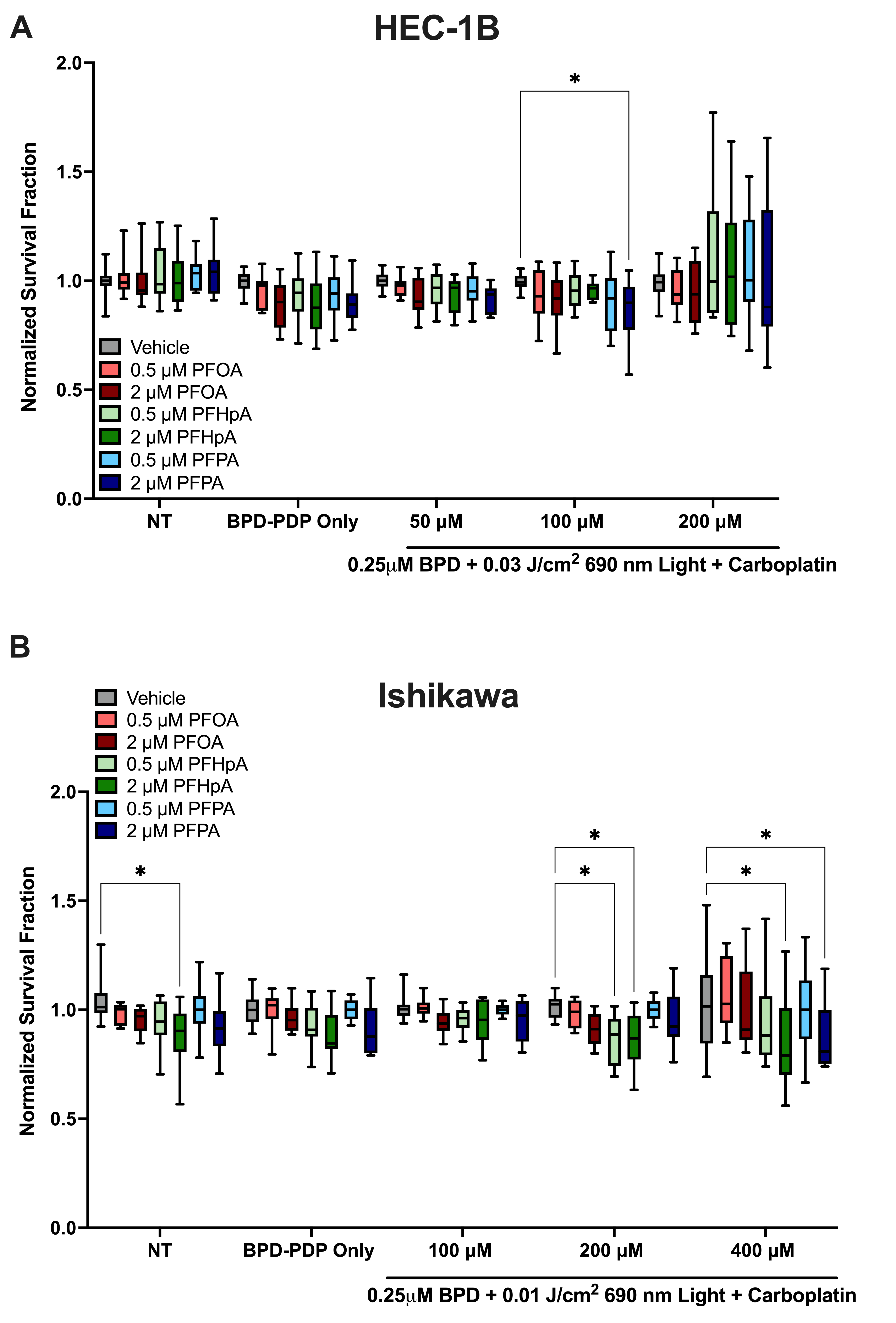


**Figure S10. Normalized survival fraction in endometrial cancer cells exposed to PFAS then treated BPD-PDP + carboplatin**. Survival fraction of PFAS-exposed A) HEC-1B and B) Ishikawa cells following BPD-PDP in combination with 50 – 400 μM carboplatin. Data shown are normalized to vehicle control at each respective carboplatin concentration and are from n= at least 5 independent experiments with two technical replicates each. Significant differences between combination therapy-treated exposure groups and exposure groups treated only with carboplatin are denoted by * (*p* < 0.05) and were determined using a two-way ANOVA with Dunnett’s test for multiple comparisons.


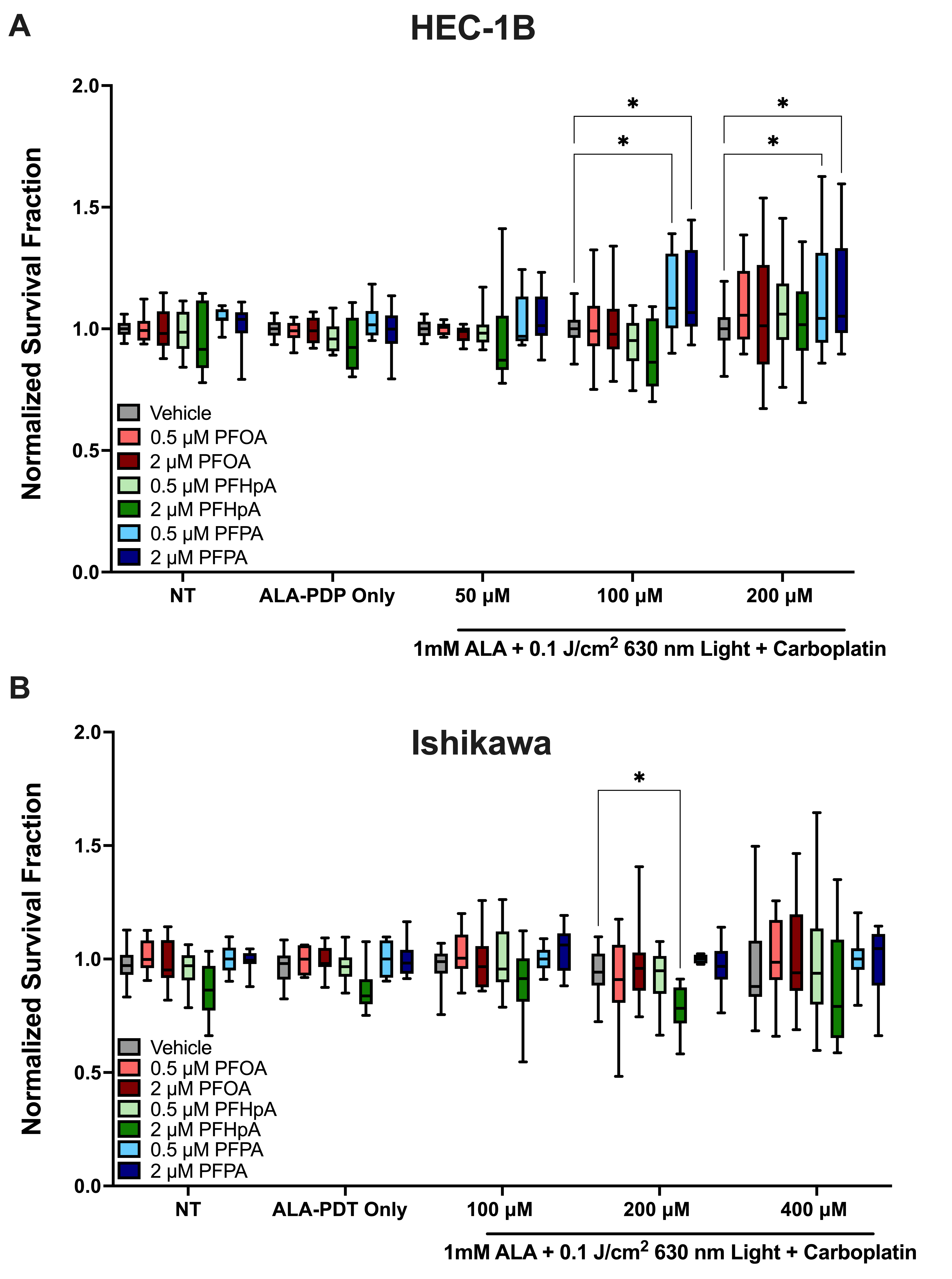


**Figure S11. Normalized survival fraction in endometrial cancer cells exposed to PFAS then treated ALA-PpIX-PDP + carboplatin**. Survival fraction of PFAS-exposed A) HEC-1B and B) Ishikawa cells following ALA-PpIX-PDP in combination with 50 – 400 μM carboplatin. Data shown are normalized to vehicle control at each respective carboplatin concentration and are from n= at least 5 independent experiments with two technical replicates each. Significant differences between combination therapy-treated exposure groups and exposure groups treated only with carboplatin are denoted by * (*p* < 0.05) and were determined using a two-way ANOVA with Dunnett’s test for multiple comparisons.


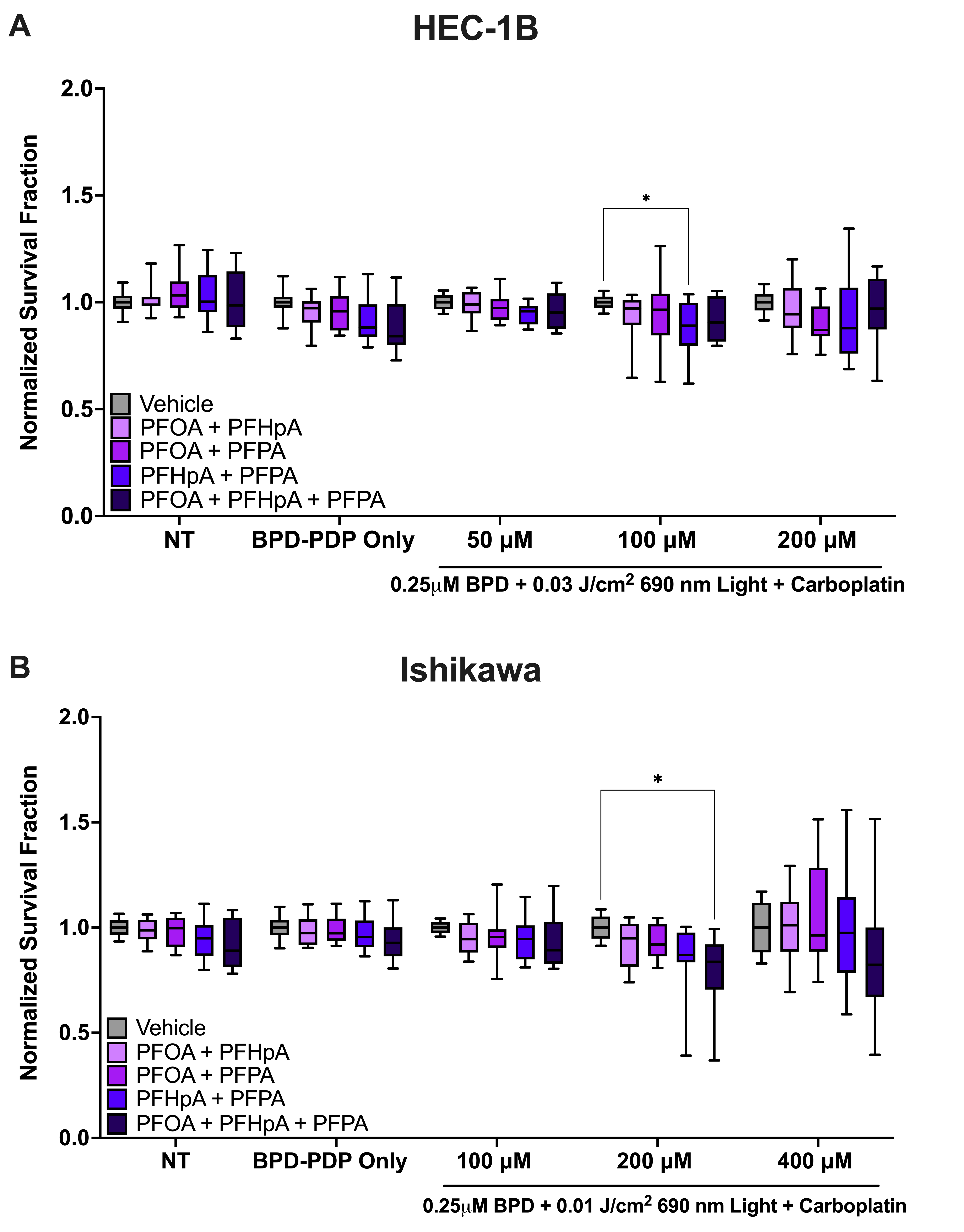


**Figure S12. Normalized survival fraction in endometrial cancer cells exposed to PFAS mixtures then treated BPD-PDP + carboplatin**. Survival fraction of PFAS mixtures-exposed A) HEC-1B and B) Ishikawa cells following BPD-PDP in combination with 50 – 400 μM carboplatin. Data shown are normalized to vehicle control at each respective carboplatin concentration and are from n= at least 5 independent experiments with two technical replicates each. Significant differences between combination therapy-treated exposure groups and exposure groups treated only with carboplatin are denoted by * (*p* < 0.05) and were determined using a two-way ANOVA with Dunnett’s test for multiple comparisons.


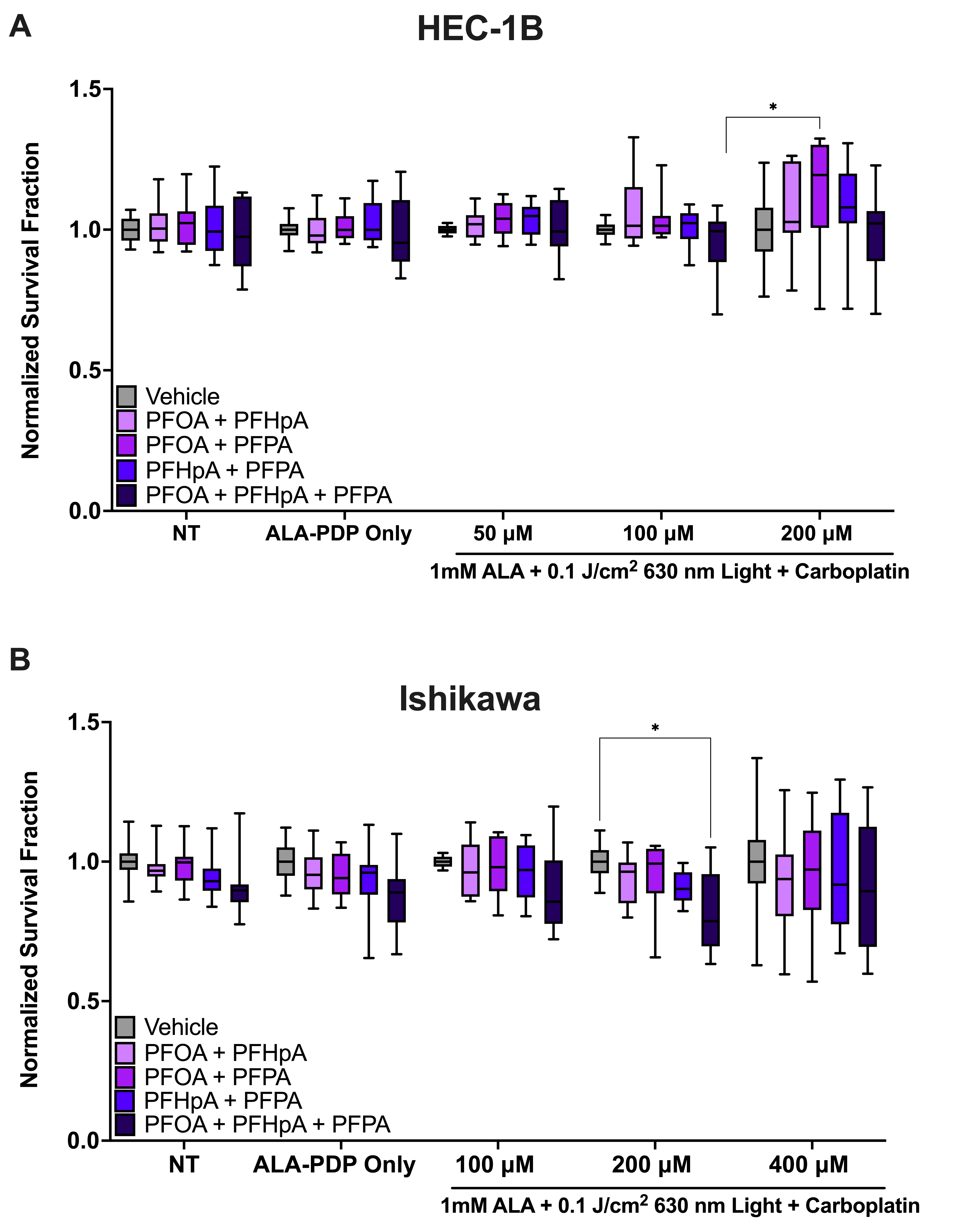


**Figure S13. Normalized survival fraction in endometrial cancer cells exposed to PFAS mixtures then treated ALA-PpIX-PDP + carboplatin**. Survival fraction of PFAS mixtures-exposed A) HEC-1B and B) Ishikawa cells following ALA-PpIX-PDP in combination with 50 – 400 μM carboplatin. Data shown are normalized to vehicle control at each respective carboplatin concentration and are from n= at least 5 independent experiments with two technical replicates each. Significant differences between combination therapy-treated exposure groups and exposure groups treated only with carboplatin are denoted by * (*p* < 0.05) and were determined using a two-way ANOVA with Dunnett’s test for multiple comparisons.

**
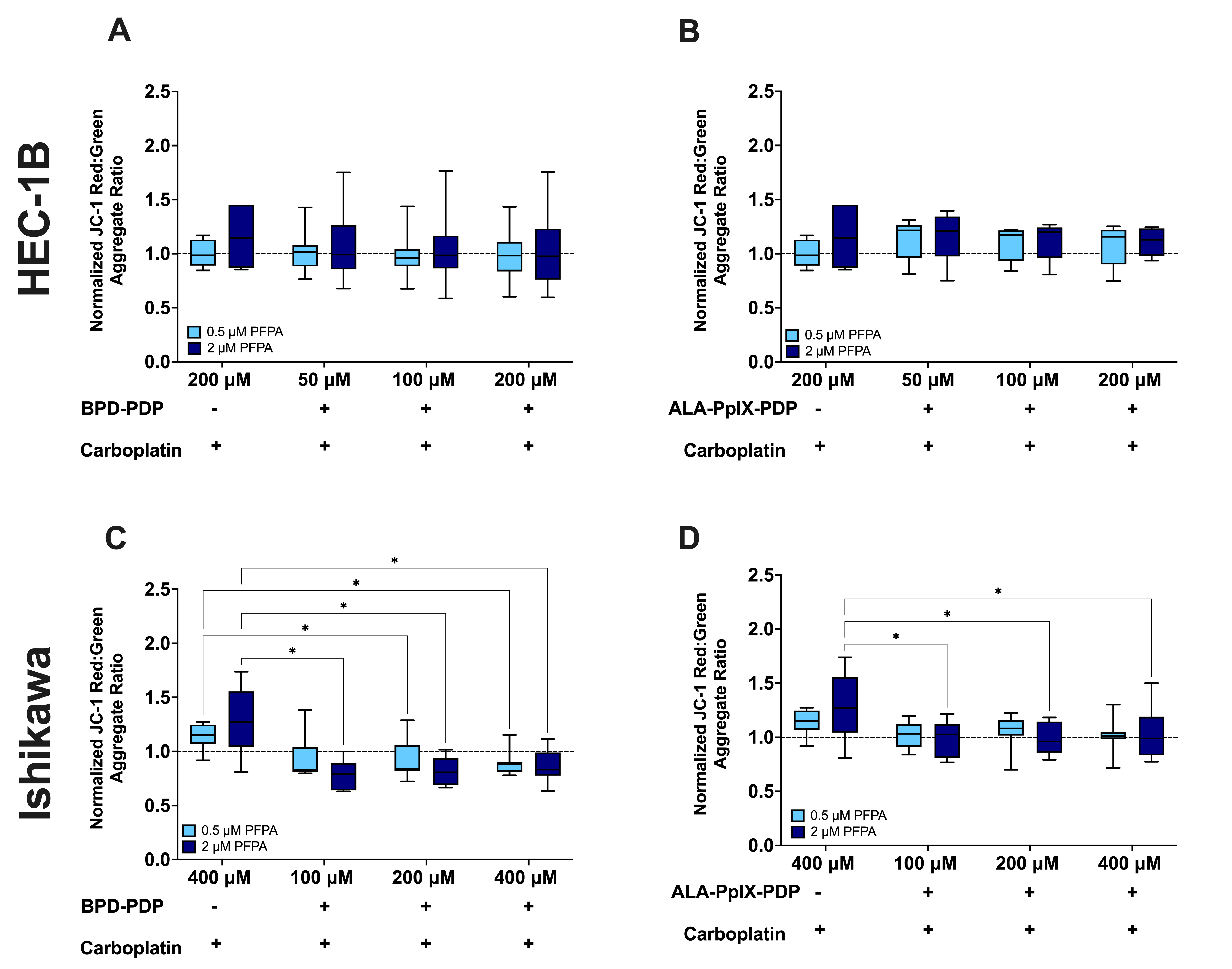
**

**Figure S14. Impact of combination therapy using either BPD-PDP (A,C) or ALA-PpIX-PDP (B,D) on ΔΨ_m_ in PFPA-exposed HEC-1B and Ishikawa cells**. Comparison of ΔΨ_m_ in HEC-1B cells that have been exposed to PFPA then treated with 200 μM carboplatin and PFPA-exposed cells treated with A) BPD-PDP (hν: 0.03 J/cm^2^) or B) ALA-PpIX-PDP (hν: 0.1 J/cm^2^) in combination with carboplatin. Comparison of ΔΨ_m_ in Ishikawa cells that have been exposed to PFPA then treated with 400 μM carboplatin and PFPA-exposed cells treated with C) BPD-PDP (hν: 0.01 J/cm^2^) or D) ALA-PpIX-PDP (hν: 0.1 J/cm^2^) in combination with carboplatin. Bars representing PFAS exposure group treated with carboplatin only represent previously published data.^1^ Data shown are normalized to their own vehicle control and are from n= at least 3 independent experiments with two technical replicates each. Significant differences between combination therapy-treated exposure groups and exposure groups treated only with carboplatin are denoted by * (*p* < 0.05) and were determined using a two-way ANOVA with Dunnett’s test for multiple comparisons.


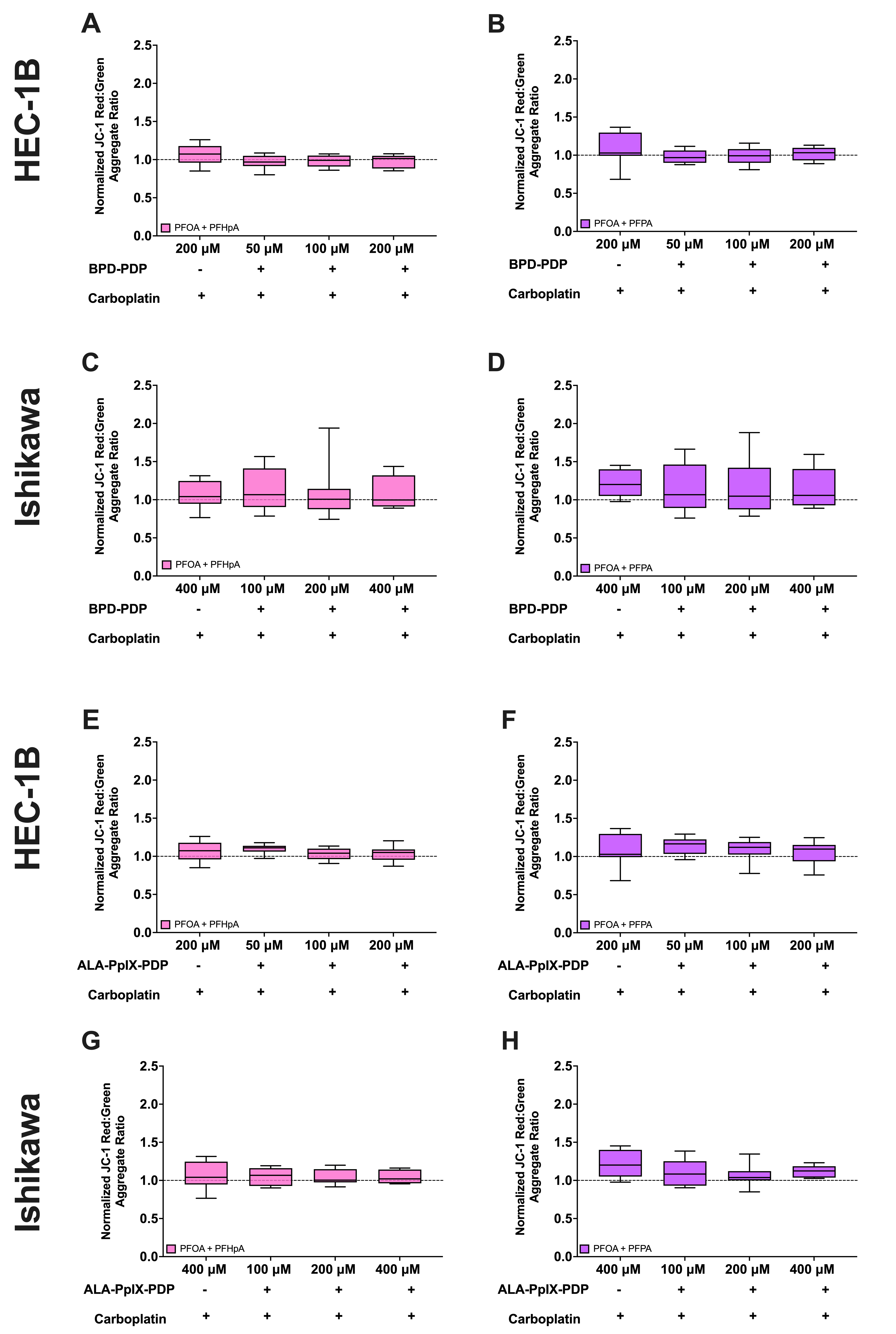


**Figure S15. Effects of combination therapy using either BPD-PDP (A-D) or ALA-PpIX-PDP (E-H) on ΔΨ_m_ in HEC-1B and Ishikawa cells exposed to alternate PFAS mixtures**. Comparison of ΔΨ_m_ in HEC-1B cells that have been exposed to PFOA + PFHpA or PFOA + PFPA then treated with only 200 μM carboplatin versus cells treated with A,B) BPD-PDP (hν: 0.03 J/cm^2^) or E,F) ALA-PpIX-PDP (hν: 0.1 J/cm^2^) in combination with carboplatin. Comparison of ΔΨ_m_ in Ishikawa cells that have been exposed to PFOA + PFHpA or PFOA + PFPA then treated with only 400 μM carboplatin versus cells treated with C,D) BPD-PDP (hν: 0.01 J/cm^2^) or G,H) ALA-PpIX-PDP (hν: 0.1 J/cm^2^) in combination with carboplatin. Bars representing PFAS exposure group treated with carboplatin only represent previously published data.^1^ Data shown are normalized to their own vehicle control and are from n= at least 4 independent experiments with two technical replicates each.


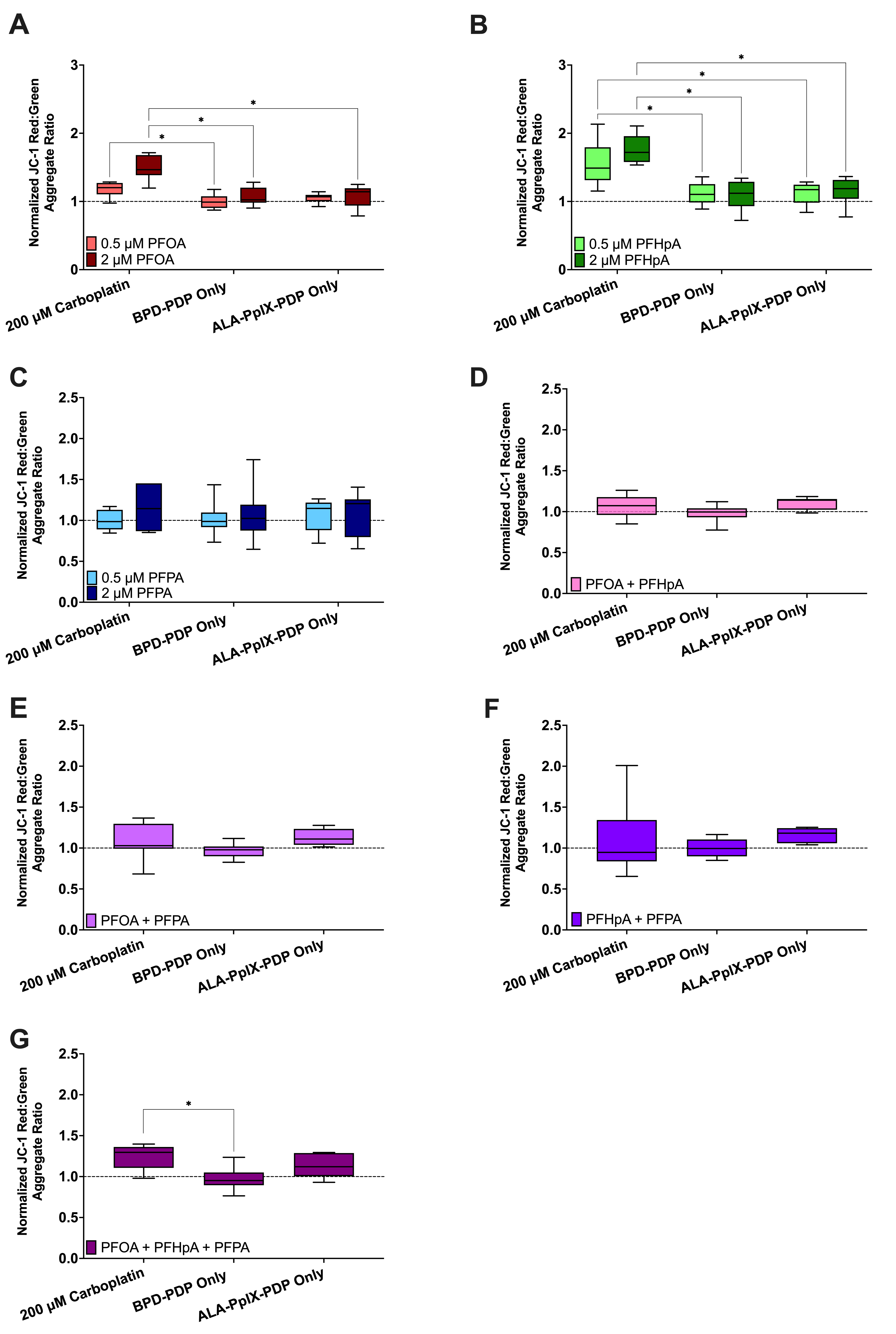


**Figure S16. Effects of BPD-PDP or ALA-PpIX-PDP only on ΔΨ_m_ in HEC-1B cells exposed to PFAS and PFAS mixtures**. Comparison of ΔΨ_m_ in HEC-1B cells that have been exposed to A-C) PFAS and D-G) PFAS mixtures then treated with only 200 μM carboplatin versus cells treated with BPD-PDP (hν: 0.03 J/cm^2^) or ALA-PpIX-PDP (hν: 0.1 J/cm^2^) only. Bars representing PFAS exposure group treated with carboplatin only represent previously published data.^1^ Data shown are normalized to their own vehicle control and are from n= at least 4 independent experiments with two technical replicates each. Significant differences between PDP-treated exposure groups and exposure groups treated only with carboplatin are denoted by * (*p* < 0.05) and were determined using unpaired t-tests or two-way ANOVA with Dunnett’s test for multiple comparisons.


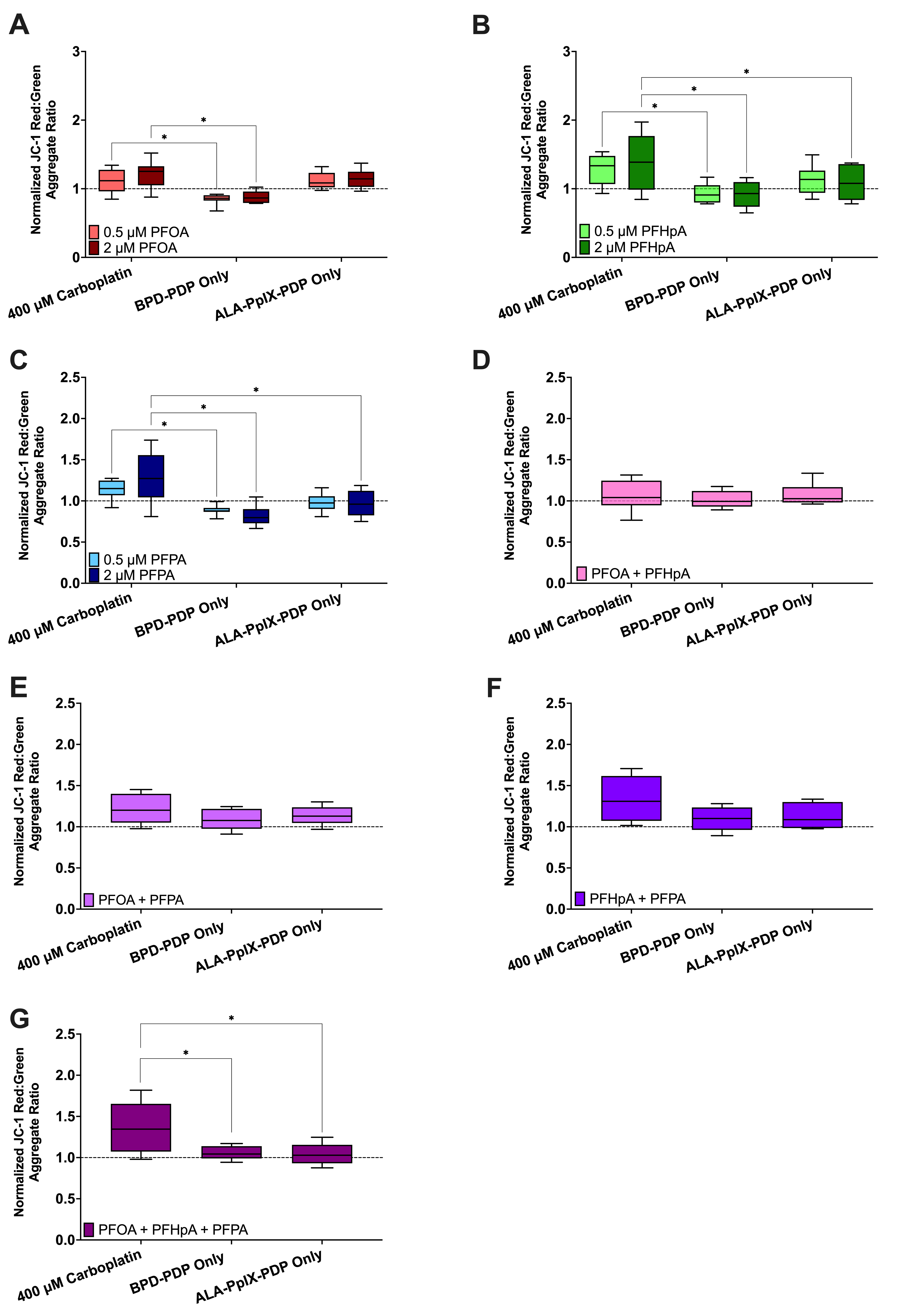


**Figure S17. Effects of BPD-PDP or ALA-PpIX-PDP only on ΔΨ_m_ in Ishikawa cells exposed to PFAS and PFAS mixtures**. Comparison of ΔΨ_m_ in Ishikawa cells that have been exposed to A-C) PFAS and D-G) PFAS mixtures then treated with only 200 μM carboplatin versus cells treated with BPD-PDP (hν: 0.01 J/cm^2^) or ALA-PpIX-PDP (hν: 0.1 J/cm^2^) only. Bars representing PFAS exposure group treated with carboplatin only represent previously published data.^1^ Data shown are normalized to their own vehicle control and are from n= at least 4 independent experiments with two technical replicates each. Significant differences between PDP-treated exposure groups and exposure groups treated only with carboplatin are denoted by * (*p* < 0.05) and were determined using unpaired t-tests or two-way ANOVA with Dunnett’s test for multiple comparisons.
